# ZNF92, an unexplored transcription factor with remarkably distinct breast cancer over-expression associated with prognosis and cell-of-origin

**DOI:** 10.1038/s41523-022-00474-2

**Published:** 2022-08-29

**Authors:** Mohammad Kamran, Udayan Bhattacharya, Mohamed Omar, Luigi Marchionni, Tan A. Ince

**Affiliations:** 1 Weill Cornell Medicine, Department of Pathology and Laboratory Medicine, New York, NY USA; 2 New York Presbyterian, Brooklyn Methodist Hospital, New York, NY USA

**Keywords:** Prognostic markers, Metastasis

## Abstract

Tumor phenotype is shaped both by transforming genomic alterations and the normal cell-of-origin. We identified a cell-of-origin associated prognostic gene expression signature, ET-9, that correlates with remarkably shorter overall and relapse free breast cancer survival, 8.7 and 6.2 years respectively. The genes associated with the ET-9 signature are regulated by histone deacetylase 7 (HDAC7) partly through ZNF92, a previously unexplored transcription factor with a single PubMed citation since its cloning in 1990s. Remarkably, ZNF92 is distinctively over-expressed in breast cancer compared to other tumor types, on a par with the breast cancer specificity of the estrogen receptor. Importantly, ET-9 signature appears to be independent of proliferation, and correlates with outcome in lymph-node positive, HER2+, post-chemotherapy and triple-negative breast cancers. These features distinguish ET-9 from existing breast cancer prognostic signatures that are generally related to proliferation and correlate with outcome in lymph-node negative, ER-positive, HER2-negative breast cancers. Our results suggest that ET-9 could be also utilized as a predictive signature to select patients for HDAC inhibitor treatment.

## Introduction

A recent pan-cancer multi-omics study concluded that cell-of-origin patterns dominate the molecular classification of 10,000 tumors from 33 types of cancer^1^. While all the cells in the body share the same DNA, the different tissue and cell types are created from the same genome by epigenetic changes involving up to a third of the entire epigenome^2,3^. Consequently, the normal cell-of-origin epigenomic profile continues to shape the tumor phenotype through interactions with transforming genetic alterations^4,5^. It has been shown that cell-origin based classification of human tumors significantly improves the taxonomy and biological understanding of breast, ovarian, pancreatic, gastric, and kidney cancers, as well as melanoma, retinoblastoma, and lymphoma^6–15^.

Many components of cellular physiology, such as gene expression, signaling, metabolism and proliferation display features of adaptive complex systems that are known to be non-linear and sensitive to initial conditions, i.e., small differences in the initial conditions may produce vastly different outcomes^16–19^. As such, it stands to reason that the normal cell-of-origin represent the most proximal and dominant initial condition for malignant transformation.

Previously, we reported an example of the cellular initial condition sensitivity in a human breast cancer model^20^. While many studies suggest that the cell-of-origin plays a role in determining tumor phenotype^21–28^, translating these results into actionable mechanisms had been difficult. We exploited a method developed by Hahn and Weinberg et al., who created the first human tumor model using completely defined genetic elements^29^. This approach allows ruling-out genetic background differences and secondary mutations as a source of phenotypic heterogeneity^20^; hence, it is particularly suited to explore epigenomic mechanisms^4,5^.

**Fig. 1:**
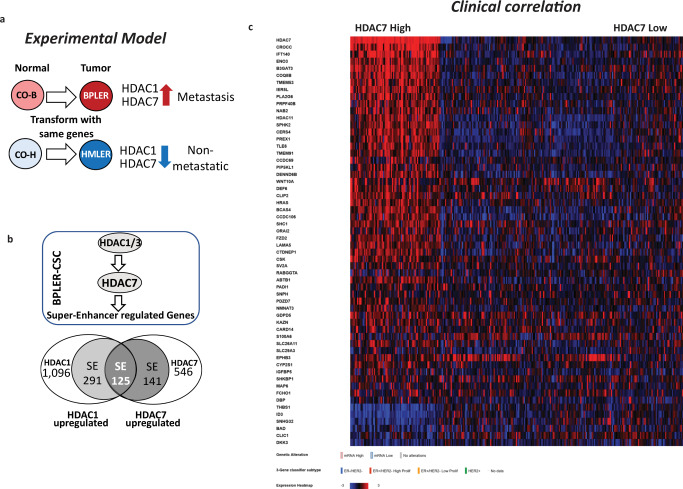
Identification of a cell-of-origin signature. **a** Two different cell-of-origins, CO-B (BPEC) and CO-H (HMEC), were isolated from the normal breast of the same donor and transformed using identical genetic elements^20^. Transformation of CO-B give rise to tumorigenic BPLER cells with high HDAC1 and HDAC7 expression. Transformation of CO-H give rise to HMLER cells with low HDAC1 and HDAC7 expression^4^. When implanted into immunocompromised mice orthotopically, BPLER cells generate invasive and metastatic xenograft tumors. In contrast, patient matched isogenic HMLER cells form indolent non-metastatic tumors^20^. **b** HDAC1/3 upregulates HDAC7, which in turn upregulates 266 super-enhancer (SE) associated genes in BPLER cells, 125 of these genes are also upregulated by HDAC1^5^. **c** The expression heatmap of 63 HDAC1/7-SE target genes that have a statistically significant correlation with HDAC7 expression in human tumors (see Supplementary Table 2 for more details). The heatmap shows that the vast majority of these genes (56/63) are over-expressed in human breast cancers with high a HDAC7 expression, consistent with the in vitro results from BPLER cells. The remaining 7 genes have an inverse correlation with HDAC7 in vivo, diverging from the in vitro results. Red = high expression, blue = low expression, (see Supplementary Fig. 1 for a high resolution of the heatmap). HDAC7 co-expression and heatmap is generated using cBioPortal online tools by analyzing METABRIC data set with complete samples (*n* = 1904)^58,59^, and mRNA expression *z*-scores relative to diploid samples^57^.

In brief, we used identical genetic elements to transform two different normal cell-of-origins (CO-B and CO-H) isolated from the same donor^20^. Implantation of these isogenic cells into mice revealed that while the CO-B derived BPLER cells formed invasive and metastatic tumors, the CO-H derived HMLER cells formed non-metastatic indolent tumors (Fig. 1a)^20^. Since the publication of these observations, several studies reported similar results confirming that transformation of different breast cell-of-origins results in distinct breast cancer phenotypes^21–23^.

The direct inheritance of pre-existing cell-of-origin features is a familiar observation where “*tumor biology will mimic the physiology of normal cell development at the time of initiation and this is preserved at least partly onwards*”^30^. For example, since the normal blood cells already travel across the body with ease, one does not use the term metastasis to describe the behavior of hematopoietic malignancies. In this case the malignant hallmark is a direct inheritance from the normal cell-of-origin.

However, reducing the cell-of-origin impact to pre-existing features excludes emergent properties associated with complex adaptive systems^31,32^. Some features may arise through a cell type specific interaction of the transformation process with the internal circuitry of the cell-of-origin. As this wiring diagram is different among various cell types, the same mutations may produce various malignant properties in some cells but not in others.

We hypothesized that certain epigenomic changes may be an emergent mechanism of propagating the cell-of-origin specific tumor phenotype^33,34^. After exploring many potential candidates, we discovered that HDAC1 and HDAC7 are upregulated during transformation in BPLER cells but not in isogenic HMLER cells^4^, and showed that HDAC7 is downstream of HDAC1/3 in regulating super-enhancer (SE) associated oncogenes through regulation of Histone 3 lysine 27 acetylation (H3K27ac)^5^. These results suggested that HDAC1/7 co-regulated genes, particularly those associated with super-enhancers in a cell-context dependent manner, may be associated with the metastatic phenotype^5^.

Since reporting these results, the role of HDAC7 in malignant phenotype has been corroborated by other laboratories in ovarian^35^, gastric^36^, lung^37^, colorectal^38^, salivary^39^, urothelial^40^, nasopharyngeal^41^ and triple-negative breast cancers (TNBC)^42^ in association with poor patient outcome, drug resistance and metastasis.

In this study we demonstrate that the downstream co-targets of HDAC1/7 include ZNF92, an unexplored transcription factor, with a single citation in the last three decades^43^. Our results indicate that the HDAC1-HDAC7-ZNF92 axis may be a compelling example of a cell context dependent emergent phenotype associated with breast cancer metastasis and survival.

## Results

### HDAC1 and HDAC7 co-regulated genes

HDAC1 and HDAC7 each regulate up to 5000 genes in different breast cancer cells^5^, making the analysis of their downstream targets challenging. However, we previously discovered that HDAC1 is upstream of HDAC7, augmenting its expression, which in turn enhances H3K27ac near super-enhancers (SEs) (Fig. 1b). Since H3K27ac is a marker of transcriptional activation, we reasoned that among the thousands of HDAC targets, the SE-associated subset upregulated in BPLER cells may be a particularly relevant category for metastasis.

Previously, we reported that HDAC1 and HDAC7 individually upregulate 1512 and 812 genes respectively in breast cancer cells^4^. Among these, only a small subset of 125 named genes are associated with super-enhancers and upregulated by HDAC1 and HDAC7 simultaneously. Henceforth, we refer to this signature as 125 gene epigenetic tumor signature (ET-125) (Fig. 1c and Supplementary Table 1)^5^.

Remarkably, almost half of the ET-125 genes in BPLER cells (56/125) demonstrate statistically significant correlation with HDAC7 expression in clinical breast cancer samples (Supplementary Table 2). Consistent with this, the mRNA expression heat-map illustrates the remarkable association of these genes with HDAC7 mRNA expression levels in human breast cancer (Fig. 1b, and Supplementary Fig. 1). Comparison of tissues vs. cultured cells is difficult due to different proliferation rates and presence of heterologous cell types in tissues such as fibroblasts, endothelium, immune cells etc. Therefore, confirmation of the in vitro ET-125 signature in human tumors to this extent was remarkable, and encouraged further exploration.

### Pathways associated with HDAC7 upregulated genes

The gene set enrichment analysis (GSEA) is a method that can assist with exploration of biological processes associated with a particular expression signature^44^. We used the Molecular Signatures Database (MSigDB) with 32,274 gene sets in nine collections (C1-8 and H) to explore the pathways that may be associated with the ET-125 signature using GSEA^44^. Among the 6290 gene sets in the MSigDB Curated gene set collection (C2), the HDAC1 targets (*p* = 2.66 e^−12^) and HDAC1/2 targets (hypergeometric *p* = 2.37 e^−10^) are identified by GSEA as the #1 and #4 ranked gene sets associated with the ET-125 signature^45^ (hypergeometric *p*-value, Supplementary Table 3a). The remarkable correlation between these independent HDAC signatures reinforces reproducibility of our results^45^.

Intriguingly, in the MSigDB Hallmark collection (H, *n* = 50), the top ten gene sets associated with the ET-125 signature in GSEA included epithelial-mesenchymal transition (*p* = 2.28 e^−7^), K-Ras signaling (*p* = 3.24 e^−6^), apoptosis (*p* = 1.52 e^−4^), Wnt-B-catenin signaling (*p* = 3.06 e^−4^), hypoxia (*p* = 4.14 e^−4^) and p53 pathway (*p* = 4.14 e^−4^) (hypergeometric *p*-value, Supplementary Table 3b). All of these pathways have been implicated in metastasis and/or poor cancer outcome; consistent with the differential expression of HDAC1/7-SE signature between metastatic BPLER vs. non-metastatic HMLER cells.

**Fig. 2:**
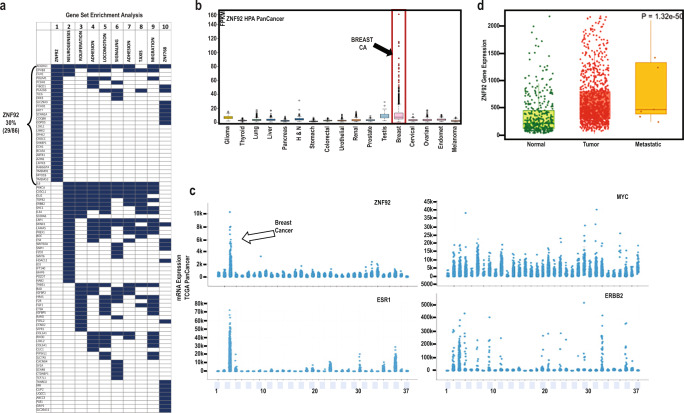
ZNF92 expression in human tumors. **a** Gene Set Enrichment Analysis (GSEA) of HDAC1/7-SE upregulated genes. The top 10 pathways are depicted in the GSEA heatmap: the blue boxes mark 86 HDAC1/7-SE upregulated genes (rows) in each column that represents a different gene set in rank order (ZNF92 first column). The *p*-value range for the top ten gene sets is 1.47e−11 to 6.5e−16 (see Supplementary Table 3 for details). The analysis is carried out using the GSEA online tool. **b** Human Protein Atlas (HPA) PanCancer expression analysis of ZNF92 RNA-seq data from 17 cancer types visualized with box plots, shown as median and 25th and 75th percentiles. Points are displayed as outliers if they are above or below 1.5 times the interquartile range. See Supplementary Table 4 for the complete list of tumor types. **c** The relative mRNA expression of ZNF92, Estrogen receptor (ERSR1), HER2 (ERBB2) and MYC in the cBioPortal TCGA PanCancer dataset that includes 37 tumor types with 10,967 samples. See Supplementary Table 5 for the complete list of 37 tumor types. Breast cancer is the third tumor type from the left. **d** The relative ZNF92 mRNA expression in tumor, normal and metastatic tissues in the TNMplot database that has RNA-seq data of TCGA including 730 normal, 9886 tumor and 394 metastatic samples^49^.

Next, we carried out a combined GSEA incorporating six MSigDB collections (C3-C8) that comprise 16,663 gene sets, including oncogenic, immunologic, cell type, regulatory and ontology gene sets. In this analysis, the top ten enriched gene sets overlapped with a majority of the ET-125 genes (86/125) (Fig. 2a, Supplementary Fig. 2, and Supplementary Table 3c).

Among the 16,663 signatures in MSigDB C3-C8, the genes containing one or more binding sites for Zinc-finger protein 92 (ZNF92) in their promoter regions (TSS −1000,+100 bp) are identified as the most significant association with the ET-125 genes (*p* = 6.5 e^−16^, Fig. 2a, hypergeometric *p*-value, Supplementary Fig. 2).

ZNFs are a large family of transcription factors that include ZNF92, which was identified in a screen of a human undifferentiated embryonal carcinoma cell line using the KRAB domain of ZNF85^46^. Among the 86 genes associated with top ten gene sets in GSEA of ET-125, 33% have ZNF92 binding sites (29/86). The remaining gene sets are associated with processes such as locomotion, adhesion, cell migration and taxis that are biological phenotypes associated with metastasis (Fig. 2a, Supplementary Table 3c).

### ZNF92 expression in breast cancer

ZNF92 is almost uniquely over-expressed in breast cancer compared to all other cancer types in the Human Protein Atlas (HPA) dataset^47^ that includes RNA-seq data from 7932 tumor samples comprising 17 cancer types (Fig. 2b, Supplementary Table 4). This result is also confirmed among the 37 cancer types represented in the TCGA PanCancer dataset that includes 10,528 tumor samples (Supplementary Table 5)^48^.

Importantly, ZNF92 over-expression appears as specific for breast cancer as estrogen receptor (ER) and HER2. In contrast, most oncogenes are typically over-expressed in multiple tumor types, similar to MYC (Fig. 2c). Additionally, using TNMplot online tools, we discovered that ZNF92 expression is increased between normal breast and breast tumors, with further increase in metastatic samples (Fig. 2d)^49^.

Interestingly, several other HDAC1/7-SE upregulated targets, such as SNPH, CCANG4, PREX1, IGFBP5, IL34 and BCAS4 also demonstrate remarkable level of breast cancer associated over-expression, providing additional support for the relevance of the ET-125 signature (Supplementary Fig. 3).

Even among the ZNF family that has been understudied in general, ZNF92 stands out as a particularly unexplored transcription factor that has never been studied in cancer since its cloning in 1993, and mentioned only once in association with the cholesterol-lowering drug atorvastatin^43,46^. Therefore, discovering the striking breast cancer specific over-expression of ZNF92 is rather unexpected.

### ET-60 and ET-9 signatures

Within the HDAC1/7-SE upregulated genes (ET-125), we identified a sixty gene subset enriched for the presence of ZNF92 binding sites, with a statistically significant correlation with in vivo HDAC7 expression, and association with patient survival. Henceforth, this subset is referred as ET-60, that includes the majority of ZNF92 targets (*n* = 22) and the genes correlating with high HDAC7 expression in vivo (*n* = 30) (Fig. 1c, Supplementary Fig. 4, Supplementary Table 6). Using the SurvExpress analysis platform^50^, we found that ET-60 identifies high, medium and low risk groups with significantly different survival hazard ratio (HR) of 5.76 (CI: 4.0–8.2) that is comparable with the 70-gene signature (Mammaprint, HR = 4.63, CI: 2.8–6.5), the 50-gene signature PAM50 (Prosignia, HR: 3.29, CI: 2.4–4.4) and a 25 gene signature BMPS (HR = 2.64, CI: 2.0–3.4), (Supplementary Fig. 5a–d)^51,52^. The ET-60 signature also correlated with metastasis, local relapse and brain relapse in NKI and SKI (GSE12276), datasets (The HR values were computed using Cox proportional hazard regression. Supplementary Fig. 5e, f). These results were similar in maximized vs. equal risk groups (Supplementary Fig. 6).

**Fig. 3:**
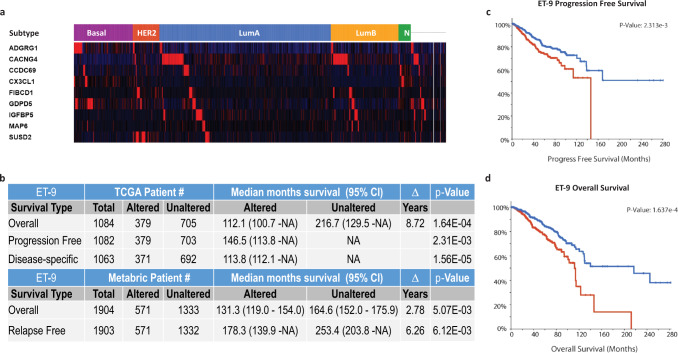
ET-9 expression and breast cancer survival. **a** The expression heatmap of ET-9 genes in the TCGA Breast Invasive Carcinoma (BIC) RNA SeqV2 dataset, including 1084 patient samples. The subtype classification is provided above the heatmap; basal-like (purple) HER2 + (red), Luminal A (blue), Luminal B (yellow), normal-like (green). **b** The relative survival statistics of breast cancer patients with altered ET-9 expression in the TCGA_BIC (*n* = 1084 patients) and METABRIC (*n* = 1904 patients) datasets^58,59^. **c** Kaplan-Meier chart of ET-9 progression-free survival in the TCGA_BIC PanCancer dataset. The ET-9 altered (red line) tumors have significantly shorter progression free survival compared to ET-9 unaltered (blue line) tumors (*p* = 0.00232, Log Rank test). **d** Kaplan-Meier chart of ET-9 overall survival in the TCGA_BIC PanCancer dataset. The ET-9 altered (red line) tumors have significantly shorter progression free survival compared to ET-9 unaltered (blue line) tumors (*p* = 0.000163, Log Rank test). All the analyses (**a**–**c**) were carried out using cBioPortal^57^.

It has been suggested that signatures with fewer genes tend to have lesser false associations, as it was reported that even random signatures of 100 genes can associate with outcome^53^. Interestingly, 23% of published breast cancer signatures showed a weaker association with outcome than the median of the random signatures of the same size^53,54^. Therefore, we used combined k-top scoring pairs (k-TSPs)^55,56^, leave-one-out single gene removal (SGR) and single gene significance analysis to identify a nine-gene subset of ET-60, henceforth referred as ET-9 (Table 1, Supplementary Fig. 8). Using cBioPortal analysis platform we examined the ET-9 signature in two datasets, the TCGA PanCancer Atlas Breast Invasive Carcinoma (PCA_BIC) and the METABRIC, with >20 years of follow up data from 1084 and 1904 patients respectively^57–59^. In the TCGA PCA_BIC the ET-9 genes are over-expressed in all subtypes of breast cancer (Fig. 3a), and ET-9 alteration is associated with progression free (*p* = 2.31e−3, Log Rank test), disease-specific (*p* = 1.56e−5, Log Rank test), and 8.7 years shorter overall survival (median 9.3 vs. 18 yrs., *p* = 1.63e−4, Log Rank test), (Fig. 3b–d)^57^. Importantly, these results are independent of clinical variables including age, ethnicity, disease stage, and radiation therapy (Supplementary Table 7). We confirmed these results in the METABRIC dataset^58,59^ where ET-9 signature is associated with a 6.2 year shorter relapse-free survival (14.9 vs. 21.1 yrs., *p* = 6.12e−3, Log Rank test) and 2.78 year shorter overall survival (*p* = 5.07e−3, Log Rank test), (Fig. 3c, d)^57^. None of the other signatures examined are associated with significant survival in both TCGA and METABRIC datasets (Supplementary Table 8).

**Table 1:** The list of genes in the ET-9 signature.

Entrez ID	ET-9 signature	Description
9289	ADGRG1 (GPR56)	Adhesion G protein-coupled receptor G1
84929	FIBCD1	Fibrinogen C domain containing 1
81544	GDPD5	Glycerophosphodiester phosphodiesterase domain containing 5
56241	SUSD2	Sushi domain containing 2
27092	CACNG4	Calcium voltage-gated channel auxiliary subunit gamma 4
6376	CX3CL1	C-X3-C motif chemokine ligand 1
3488	IGFBP5	insulin like growth factor binding protein 5
4135	MAP6	microtubule associated protein 6
26112	CCDC69	coiled-coil domain containing 69

**Fig. 4:**
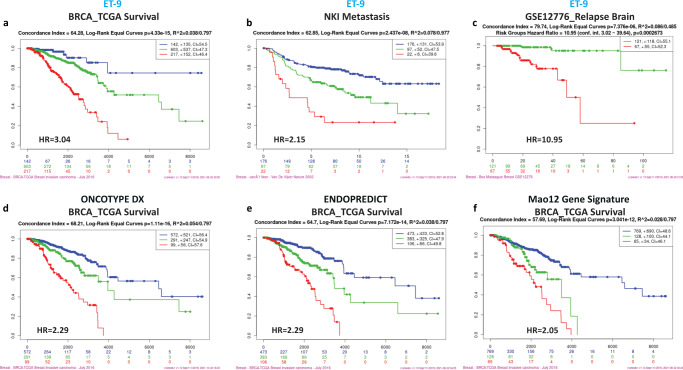
ET-9 prognostic groups in three different datasets. The Kaplan-Meier (KM) overall survival charts of human breast cancer generated using SurvExpress; high risk (red line), medium risk (green line), low risk (blue line) groups, with auto-selection of cut-off values and maximized risk groups. The relative hazard ratios (HR) were computed using Cox proportional hazard regression analysis^50^. **a** KM chart of ET-9 expression in human breast cancer in BRCA_TCGA 2016 dataset, HR: 3.04 (CI: 2.2–4.1). **b** KM chart of ET-9 expression in human breast cancer in NKI dataset, HR: 2.15 (CI: 1.6–2.8). **c** KM chart of ET-9 expression in human breast cancer in GSE12276 dataset, HR: 10.95 (CI: 3.0–39.6). **d** KM chart of the 21-gene Oncotype in BRCA_TCGA 2016 dataset, HR: 2.29 (CI: 1.61–2.97). Two genes TRFC and RPLPO not found in this dataset. **e** KM chart of a 12-gene signature (Endopredict) in BRCA_TCGA 2016 dataset, HR: 2.29 (CI 1.8–2.8). **f** KM chart of a 12-gene signature^62^ in BRCA_TCGA 2016 dataset, HR: 2.05 (CI 1.6–2.5).

Next, we confirmed these results in three additional datasets using the ServExpress analysis platform^50^, where the ET-9 expression signature identified high, medium and low risk groups with significantly different overall survival in TCGA_BRCA_2016 dataset (HR = 3.04), comparable with Oncotype^60^, Endopredict^61^ and another 12 gene signature^62^. Consistent with the derivation of ET-9 and ET-60 in a metastatic model, both signatures correlate with metastasis in the NKI dataset and brain relapse in the GSE12276 dataset (Fig. 4 and Supplementary Fig. 4).

### Tumor subtype, stage and proliferation signature

Multigene signatures generate valuable prognostic information for the subset of breast cancer patients where clinical, histopathological and immunohistochemical markers do not provide adequate guidance^63^.

Currently, the Oncotype, Progsignia, Mammaprint and Endopredict signatures are recommended generally for early-stage, ER-positive, HER2-negative, and lymph node negative breast cancers^64,65^. In addition, the Mammaprint signature is recommended for breast cancers with up to 3 metastatic lymph nodes (N1)^66,67^. Accordingly, further development of molecular prognostic tests for the remaining patient populations such as ER-negative, HER2-positive and late stage metastatic or treated breast cancers would be beneficial^60,61^.

**Fig. 5:**
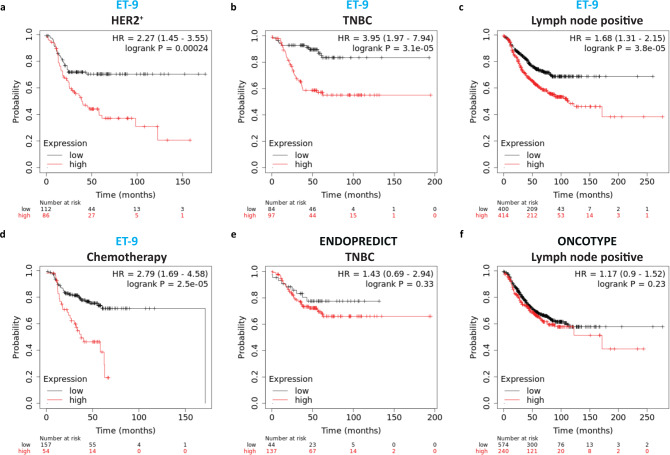
ET-9 in breast cancer subgroups. The Kaplan-Meier (KM) charts of relapse free survival of human breast cancer generated using Kaplan-Meier plotter [Breast] high risk (red line), low risk (black line). The analysis is carried out by using all probe sets per gene with auto selection of best cut off values, exclusion of biased arrays, and no data censoring and multivariate analysis. The relative hazard ratios (HR) were computed using Cox proportional hazard regression analysis^68^. **a** KM chart of ET-9 in HER2 + human breast cancer, HR: 2.27 [CI 1.45–3.55], *p* = 2.4e−4. **b** KM chart of ET-9 in triple negative breast cancer (TNBC), HR: 3.95 [CI 1.97–7.94], *p* = 3.1e−5. **c** KM chart of ET-9 in lymph node positive human breast cancer, HR: 1.68 [CI 1.31–2.15], *p* = 3.8e−5. **d** KM chart of ET-9 in breast cancer patients with systemic chemotherapy, HR: 2.79 [CI 1.69–4.58], *p* = 2.5e−5. **e** KM chart of 12-gene Endopredict signature in TNBC, HR: 1.43 [CI 0.69–2.94], *p* = 0.33. **f** KM chart of Oncotype DX in lymph node positive human breast cancer, HR: 1.17 [CI 0.9–1.52], *p* = 0.23.

We examined ET-9 signature using K-M plotter^68^ and show that high ET-9 expression is associated with shorter survival in lymph node positive (HR = 1.6, CI 1.3–2.1, *p* = 3.8e−5), HER2 positive (HR = 2.2, CI 1.4–3.5, *p* = 2.3e−4), post-chemotherapy (HR = 2.7, CI 1.6–4.5, *p* = 2.5e−5,) and triple-negative breast cancers (HR = 3.9, CI 1.9–7.9, *p* = 3.1e−5). The HR values were computed using Cox proportional hazard regression. (Fig. 5a–d). These results were similar for ET-60 (Supplementary Fig. 5), and suggest these signatures may have an additive or complimentary utility with other prognostic signatures (Fig. 5e, f, Supplementary Fig. 7e, f)^69^. It is worth mentioning that these results were not sensitive to changing cut-off points (Supplementary Fig. 9) and the ET-125, ET-60 and ET-9 signatures were comparable in their prognostic power, particularly for basal-like, lymph-node positive and chemotherapy treated patients where Oncotype Dx was not prognostic (Supplementary Fig. 10).

One common feature of most breast cancer prognostic tests is their apparent association with proliferation signatures (Supplementary Table 9)^52^. It was reported that proliferation associated genes are over-represented in 22 out of 24 breast prognostic signatures^70^, which is partially redundant with histological grading that incorporates mitotic counts^71,72^. Consistent with this, removal of proliferation associated genes (*n* = 131) in 47 published breast cancer prognostic signatures, reduced their association with outcome decreased dramatically in another study^53^.

We found no overlap between ET-9 and ET-60 with the 131 gene proliferation signature^53^. Therefore, there was no reduction in HR with this adjustment. These results suggest that there are opportunities to improve prognostic signatures independent of proliferation.

**Fig. 6:**
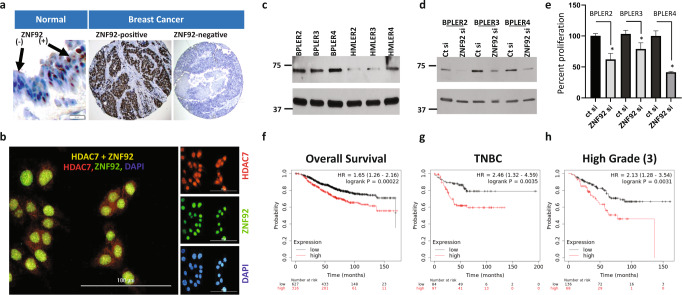
ZNF92 expression and function. **a** Immunohistochemical stain of formalin fixed paraffin embedded sections probed with ZNF92 antibody. The normal breast demonstrates two subgroups of nuclear ZNF92-positive and ZNF92-negative cells (left panel, scale bar = 20 uM). In some breast cancers nearly all tumor cells are ZNF92-positive (middle panel) and other tumors are entirely ZNF92-negative (right panel, scale bar = 100 uM). **b** Immunofluorescent staining of HDAC7 (red), ZNF92 (green) and nuclear DAPI (blue) in BT20 cells. The merged panel demonstrates the co-expression of HDAC7 and ZNF92 in the same nuclei. The brightness of the entire digital image was increased in the single channel panels to visualize the lower cytoplasmic staining and the individual color channels were adjusted in the merged image for clarity. The uncropped and unprocessed images are provided in Supplementary Fig. 13. **c** Western blot analysis of ZNF92 protein expression in matched pairs of BPLER/HMLER 2, 3 and 4. **d** Western blot analysis of ZNF92 protein after siRNA knock-down of ZNF92 expression (ZNF92 si) in three independent BPLER lines compared to control siRNA (ct si). The uncropped and unprocessed western blot images are provided in Supplementary Fig. 14. **e** Bar graphs showing that siRNA knock-down of ZNF92 expression (ZNF92 si) results in statistically significant reduction (*) in cell numbers in all three independent BPLER lines compared to control siRNA (ct si). **f**–**h** KM-plot survival analysis of the 29 genes in ET-125 with a ZNF92 binding site, demonstrating a correlation between high expression of ZNF92 targets and poor overall survival in all breast cancers (HR = 1.6, *p* = 0.0002) and high-grade (grade 3) breast cancer (HR = 2.1, *p* = 0.003), as well as relapse free survival in triple-negative breast carcinoma (HR = 2.4, *p* = 0003) The relative hazard ratios (HR) were computed using Cox proportional hazard regression analysis. See Supplementary Fig. 11 for overall and relapse free survival for other breast cancer cohorts including HER2, Basal-like, Luminal-B, and chemotherapy treated cohorts.

Lastly, we demonstrate that normal human breast luminal epithelium is composed of two subtypes of cells; those that are ZNF92 protein positive and others that are ZNF92 negative (Fig. 6a). Consistent with this cell-origin pattern, we observed that some human breast cancers are strongly ZNF92 protein positive, and others are almost entirely ZNF92 negative (Fig. 6a). All the breast cancer cell lines we tested indicate that ZNF92 protein is co-expressed with HDAC7 in the nucleus (Fig. 6b). Consistent with the derivation of the ET-125 signature in a differential screen of patient matched metastatic BPLER vs. non-metastatic HMLER cells, we found that BPLER cells express higher levels of ZNF92 (Fig. 6c). In addition, we found that knock-down of ZNF92 inhibits cell proliferation (Fig. 6d, e) and cell migration (Supplementary Fig. 11). We also found that the higher expression levels of the twenty-nine ET-125 genes that contain ZNF92 binding sites correlate with overall and relapse-free survival in HER2+, Luminal-B, TNBC, and basal-like breast cancers independent of grade and treatment (Fig. 6f–h, Supplementary Fig. 12).

These data indicate that the subset of poorly-differentiated high-grade breast cancers with elevated ZNF92 signature are almost two fold more likely to relapse compared to other high grade breast cancers with lower ZNF92 signature (HR = 1.9, *p* = 0.0006) (Supplementary Fig. 12). Likewise, even among patients who received systemic chemotherapy, the subset of tumors with higher ZNF92 signature expression were 2.8-fold more likely to relapse, compared to those with a lower ZNF92 signature (*p* = 0.000024). The HR values were computed using Cox proportional hazard regression (Supplementary Fig. 12).

## Discussion

Some of the genes in the ET-9 signature have been previously associated with breast cancer outcome. The over-expression of insulin like growth factor binding protein 5 (IGFBP5) correlates with poor prognosis^73^ and lymph node metastasis^74^. In addition, genetic variations in IGFBP5 are associated with increased breast cancer risk in African-American patients^75^.

The C-X3-C Motif Chemokine Ligand 1 (CX3CL1 or Fractalkine) is a multifunctional inflammatory chemokine. While the transmembrane form of CX3CL1 is an adhesion molecule, the soluble form functions as a chemotactic cytokine. CX3CL1 is associated with metastasis and poor prognosis in breast cancer^76–78^.

The normal L-type voltage-gated calcium channel gamma subunit (CACNG4) regulates the trafficking and gating of AMPA-selective glutamate receptors. In breast cancer, CACNG4 is upregulated in lymph node metastasis and associated with poor prognosis^79^. Experimentally, CACNG4 has been associated with cell motility, transformation and metastasis^79^.

The adhesion G-protein–coupled receptor G1 (ADGRG1/GPR56) is associated with cell-cell and cell-matrix interactions, and implicated in bone metastasis in breast cancer^80^. The Sushi Domain Containing 2 (SUSD2) is a cell membrane protein with adhesion domains that interacts with Galectin-1 to promote breast cancer immune evasion, angiogenesis, invasion and metastasis^81^. The Glycerophosphodiester Phosphodiesterase Domain Containing 5 (GDPD5) protein is involved in lipid metabolism. It was found that GDPD5 knock-down inhibits breast cancer cell proliferation, migration, and invasion^82^.

While the other ET-9 genes have not been studied in breast cancer, they have been implicated in other cancers. For example, overexpression of Fibrinogen C domain containing 1 (FIBCD1), which is a transmembrane endocytic receptor, correlates with poor prognosis in gastric and liver cancers^83,84^. Microtubule Associated Protein 6 (MAP6) Domain Containing 1 protein is implicated in lymph node metastasis in prostate cancer^85^, and the over-expression of Coiled-Coil Domain Containing 69 (CCDC69), which is scaffold protein involved in DNA replication and mitotic spindle formation, is associated with cisplatin-resistance ovarian cancer cells^86^.

In sum, the ET-9 signature contains a chemokine, a calcium channel subunit, a G-protein-coupled receptor, a membrane adhesion protein, a lipid phosphodiesterase, an endocytic receptor, a microtubule associated protein and a scaffold protein. This collection of genes appears different than signatures that are typically enriched for oncogenes, growth factors, and cell cycle proteins. While we know that these nine genes are downregulated with HDAC7 knock-down and three of them are ZNF92 targets (FIBCD1, GDPD5, and GPR56/ADGRG1), they have not been extensively studied in cancer; a PubMed search with the keyword cancer returned less than twenty publications for FIBCD1 (*n* = 7), CACNG4 (*n* = 7), CCDC69 (*n* = 8), MAP6 (*n* = 11) and GDPD5 (*n* = 19). Moreover, it is not known whether enzymatic or non-enzymatic activities of HDAC7 is involved in the regulation of these genes. Therefore, understanding the combined function of these ET-9 genes and their regulation by HDAC7 and ZNF92 will require further investigation.

In this study we describe a *cell-of-origin emergent phenotype* in human breast cancers with a significant impact on patient survival. Our hypothesis-based approach, focusing on downstream targets of HDAC1/7-SE (ET-125), resulted in gene expression signatures that do not overlap with omics-derived or proliferation signatures. The ET-9 signature is independent of patient age, ethnicity, disease stage and proliferation, and correlates with patient outcome in triple-negative breast cancer, HER2+, lymph node positive, chemotherapy treated, and brain metastatic breast cancers. In addition, we identified ZNF92, a transcription factor that has never been studied in cancer, as a marker that is almost uniquely over-expressed in human breast cancer.

Previously we described a *cell-of-origin inherited phenotype* spectrum in human breast tumors with significant impact on survival^12^. In brief, in normal human breast tissue we identified eleven normal cell types each with a distinct methylation profile^11,87,88^. Both the marker profile and methylation patterns of these normal cell types are preserved in human breast cancers^11,87,88^. Hence, each human breast tumor resembles one of the eleven normal cell types that can be grouped into 4 major subtypes, HR0–HR3, based on vitamin D, androgen, and estrogen hormone receptor (HR) expression^11^. Importantly, there is a nearly seven-fold survival difference between HR0 (ER/AR/VDR negative) vs. HR3 (ER/AR/VDR positive) tumors, indicating a strong cell-of-origin influence in human breast cancer^11^.

Since our initial report, multiple studies showed that cancer epigenomes are dominated by patterns already present in the normal cell-of-origin, and correlated with patient survival in cholangiocarcinoma, leukemia, brain and lung tumors^2,3^. Likewise, it was found that cell-of-origin patterns dominate the molecular classification of 10,000 tumors from 33 types of cancer^1^. Cumulatively, these results indicate that both *inherited and emergent* cell-of-origin features can have a significant impact on human breast cancer behavior. The cell-origin associated signatures can be utilized as prognostic tests, as well as predictive tests to select patients for AR, VDR and HDAC targeted therapies.

The direct analysis of human tissues with omics approaches have identified clinically relevant prognostic signatures. However, retroactively assigning a mechanistic meaning to these signatures is not always possible, it was recently found that that none of the 48 previously published breast cancer prognostic signatures has a sensible biological interpretation or meaning with respect to disease etiology^89^. Moreover, other studies found that there is up to 60% risk assignment discordance between Oncotype DX, PAM50 and Mammaprint, classifying the same sample as low risk in one assay and high risk for another^52^.

In contrast, experimental models do provide mechanistic signatures; however, these are not always relevant in vivo. In light of this, it is worth mentioning that the BPLER model we used in this study has been validated multiple studies during the past two decades, indicating that the signatures derived from this model appear to have clinical relevance^4,20,90–98^. It may be possible to improve on this model by blending signatures that represent distinct hallmarks of cancer such as proliferation, apoptosis, angiogenesis, inflammation, immune response, and mutational burden^99,100^.

Cumulatively, our results indicate that cancer is a complex system where the behavior of the entire system is more than the simple sum of its parts. These findings caution against classification and treatment of human tumors simply based on genetic alterations without considering the cell-origin context, particularly since the same genes produce different phenotypes in different cells^20,101–103^.

## Methods

### Cell lines

The BPLER and HMLER cells were established previously^20^ and characterized extensively^4,20,90–98^. The BPLER cells are cultured in the BMI-T medium (US Biological, cat# 506387.500, or TumoriGenesis Product No: 833)^5^, and the HMLER cells are cultured in MEGM medium (Lonza,cat# CC-3150)^4,20^. The BPLER and HMLER cells we established were tested for mycoplasma and deposited to ATCC (American Type Culture Collection, ATCC item #s CRL-3546 and CRL-3547) and European Collection of Authenticated Cell Cultures (ECACC, Accession numbers; 20012030, 20012033, 20012038, 20012041, 20012044, and 20012047, https://www.ukbrcn.org/news/new-accessions-coming-soon-to-the-european-collection-of-authenticated-cell-cultures/).

### Immunohistochemistry

The deparafinized slides were treated with Sodium Citrate Buffer (10 mM Sodium Citrate, 0.05% Tween 20, pH 6.0) at 98 °C for 20 min in a scientific microwave to achieve heat-induced epitope retrieval. Next, these slides were blocked in 0.3% H2O2 in TBS for 15 min. to prevent non-specific binding. Primary ZNF92 antibody (ab170885) was diluted (1:100) in Dako antibody diluent, and applied over the tissue overnight at 4 °C in humidified chamber, and developed with chromogen for 5–10 min at room temperature (Dako K3467 kit).

### Immunofluorescence assay

BT20 cells were seeded at 8 well Lab-Tek^R^ II Chamber Slide^TM^ glass slide, 24 hours prior to staining. eBioscience™ Foxp3/Transcription Factor Staining Buffer Set (Life technologies-00–5523–00) was used for staining, nuclear staining protocol was followed. Anti HDAC7 (ab12174) and anti ZNF92 mouse (Life technologies-MA524530) antibodies were used in 1:500 dilution overnight at 40 C. Anti-Rabbit-AlexaFlour 647 (Life technologies-A32733) and Anti-Mouse-AlexaFlour 488 (Life technologies-A11001) were used in 1:500 dilution for secondary staining at room temperature for 30 min. Imaging was performed at Lionheart™ FX Automated Microscope by BioTek at 40X magnification. The brightness of the entire image was increased in Fig. 6b, the unprocessed images are provided in the Supplementary Fig. 13.

### ZNF92 knockdown and scratch assay

BT20 cells were seeded in 6-well plates, 24 hours prior to knockdown (KD). Next day, scratch was made using P1000 pipette tip, followed by KD. Migration of cells was monitored by taking images at Leica DMI1 inverted microscope equipped with LEICA MCHD120 camera. Healing/migration was quantified by ImageJ area tool. 50 nM Control si (Sigma Aldrich-SIC001), ZNF92 si1, si2 and si3 (Ambion-AM16708-ID238043, 110198, 110199) were used for KD. KD was performed twice, 48 hours apart. Lipofectamine RNAiMAX Reagent was used as a transfection reagent. Pooled si (50 nm each) was used for BPLERs. Transfection was performed as per the recommended protocol.

### Western blot analysis

Cell pellets were collected, washed twice with PBS and frozen in −80 freezer prior to lysis. Lysis was performed in 1XRIPA lysis buffer (Millipore 20–188) supplemented with Halt Protease and Phosphatase Inhibitor cocktail (Thermofisher Scientific 78442), 1 mM PMSF (Millipore Sigma P7626), 10 mM Sodium OrthoVanadate (Millipore Sigma S6508), 1X cOmplete, Mini, Protease Inhibitor Cocktail (Roche 11836153001) and 1X PhosSTOP EASYpack Phosphatase Inhibitor Cocktail (Roche 04906837001). Cell lysate was prepared in 1X Laemmli sample buffer (Bio-Rad-1610737). 30 micrograms cell lysate was separated in 4–15%-Mini-PROTEAN TGX Precast Protein Gel (BioRad Laboratories 4561084). Trans-Blot Turbo Mini PVDF Transfer Packs (BioRad Laboratories 1704156EDU) and Trans-Blot Turbo Transfer system (BioRad laboratories) was used to perform transfer of protein. Blots were blocked in 5% skimmed milk (SignaGen Laboratories SL100317) with 1XTBST (VWR Life Science K873). Detection of protein was performed by Western blotting using specific antibodies against ZNF92 (ab170885), Vimentin (ab54373), and Beta actin (Sigma A2228). HRP labeled secondary anti-mouse and anti-rabbit (PIERCE 31430, 31402) were used to detect bands. SuperSignal West Dura Extended Duration (Thermofisher Scientific 34076) substrate was used to develop the blots. The uncropped and unprocessed images are provided in Supplementary Figs. 14, 15.

### Gene set enrichment analysis

The list of gene identifiers is entered in the box provided in the investigate gene sets tab and Homo sapiens species is selected. Compute overlap is selected for the relevant MSigDB collections, with false discovery rate (FDR) *q*-value less than 0.05. The *p* values in GSEA are based on Hypergeometric test.

### Survival analysis

The SurvExpress analysis was carried out selecting; (a) not censored for survival days, (b) without stratification, (c) heat map by prognostic index, (d) network none, (e) no imputation, (f) no quantization, (g) advanced check, (h) attribute plot check, (g) maximized risk groups, and with default options for other variables. Depending on the analysis we selected two or three risk groups, determined by prognostic index (risk score) estimated by beta coefficients multiplied by gene expression values. The risk group splitting is optimized using an algorithm that decides where the partitions should be made to maximize the statistical significance of the separation of risk groups as described. In brief, the ‘Maximize Risk Groups’ option in SurvExpress uses and algorithm that tests different cut-off points until it partitions the risk groups with the minimum *p*‐value. The *p* values were computed using Log Rank test, and the relative hazard was computed using Cox proportional hazard regression analysis^50^.

The Kaplan-Meier Plotter analysis was carried out selecting the following parameters; split patients by auto select best cutoff (checked), survival (RFS or OS); follow up threshold (all), censor at threshold (unchecked), compute median over entire database (false), probe set option (user selected probe set), invert HR values below 1 (no). We tested several alternative approaches available to define comparison cohorts (a) quantile cut-off at the median, upper, and lower quartiles, (b) trichotomizing (T1 vs. T3 or Q1 vs Q4) which involves assigning the data into three cohorts and then omitting the middle cohort, or (c) using the best available cut-off value where each possible cutoff value is tested between the lower and upper quartiles, and False-Discovery Rate using the Benjamini-Hochberg method is used to correct for multiple hypothesis testing. The results shown are with the best available cut-off value. However, it is possible to generate similar results using the quantile and trichotomizing approaches in some breast cancer subsets (Supplementary Fig. 9). The *p* values were computed using Cox proportional hazard regression analysis and false-discovery rate was computed using the Benjamini-Hochberg method to correct for multiple hypothesis testing^68^.

The following parameters were selected for CBioPortal analysis: (a) Study (Breast Invasive Carcinoma TCGA PanCancer), genomic profile (mRNA expression *z*-scores relative to diploid samples RNA Seq V2 RSEM), patient set (all samples, *n* = 1084), gene list (user-defined). (b) Study (Breast Cancer METABRIC, Nature 2012 & Nat Commun 2016); genomic profile (mRNA expression *z*-scores relative to diploid samples, RNA Seq V2 RSEM), patient set (complete samples, *n* = 1904), gene list (user-defined). In cBioPortal, the *p*-values were computed using Log Rank test, and the *q*-values were computed using the Benjamini-Hochberg false discovery rate procedure^57^.

The TNM Plot dataset includes 56,938 unique multilevel quality controlled samples: Genechip from GEO: 3691 normal, 29,376 tumor and 453 metastasis, RNA-seq from GTex: 11,215 normal, RNA-seq from TCGA: 730 normal, 9886 tumor and 394 metastasis, RNA-seq from TARGET: 12 normal, 1180 tumor and 1 metastasis^49^.

Several approaches were used to for the identification of the smallest prognostic subset of the ET-125 signature. (a) Single gene removal (SGR), where each gene is categorized as *‘non-altered’* if the expression value of each gene is between −2 and 2-fold relative to diploid samples, otherwise it is categorized as ‘*altered’*. In a step-wise manner, one gene is removed and the association of the remaining sets of genes with the overall survival is computed using Kaplan-Meier plot. The set of genes with the lowest *p*-value are selected and the steps mentioned above are repeated. (b) K-top scoring pairs (k-TSPs) analysis was carried out as previously described^56^. In brief, the k-TSPs is a rank-based algorithm which selects gene pairs whose orders changes consistently between the two classes of interest. Hence, k-TSPs is not sensitive to data preprocessing and normalization. The SGR and k-TSPs approaches identified CCDC69, CX3CL1, GDPD5, IGFBP5, CACNG4, FIBCD1, and MAP6 in the Metabric dataset. We found that SUSD2 and ADGRG1 are prognostic as single markers. We found that combining these nine genes was able to replicate the prognostic significance and robustness of larger ET signatures (Supplementary Figs. 8, 10).

### TCGA PanCancer analysis

RNA-seq data from 17 cancer types representing 21 cancer subtypes with a corresponding major cancer type in the Human Pathology Atlas^48^. The TCGA RNA-seq data was mapped using the Ensembl gene id available from TCGA, and the FPKMs (number Fragments Per Kilobase of exon per Million reads) for each gene were subsequently used for quantification of expression with a detection threshold of 1 FPKM. RNA cancer tissue category is calculated based on mRNA expression levels across all 17 cancer tissues and include: cancer tissue enriched, cancer group enriched, cancer tissue enhanced, expressed in all, mixed and not detected. Normal distribution across the dataset is visualized with box plots, shown as median and 25th and 75th percentiles. Points are displayed as outliers if they are above or below 1.5 times the interquartile range^48^.

### Reporting summary

Further information on research design is available in the Nature Research Reporting Summary linked to this article.

## Supplementary information

Supplementary Material

Reporting Summary

## Data Availability

The datasets generated during and/or analyzed during the current study are available online; see GSE131631, GSE131632, cBioPortal https://www.cbioportal.org/; GSEA https://www.gsea-msigdb.org/gsea/index.jsp; KM plotter https://kmplot.com/analysis/; TNM plot https://tnmplot.com/analysis/; Human Protein Atlas, https://www.proteinatlas.org/; SurvExpress http://bioinformatica.mty.itesm.mx/SurvExpress; and GENT2 http://gent2.appex.kr/gent2/.
