# Supplementary material for: ZNF92, an unexplored transcription factor with remarkably distinct breast cancer over-expression associated with prognosis and cell-of-origin
Source: NPJ Breast Cancer. 2022 Aug 29;8:99. doi: 10.1038/s41523-022-00474-2 (PMC9424319; doi:10.1038/s41523-022-00474-2)
Supplement: Supplementary file 1 — Supplementary Material [file 41523_2022_474_MOESM1_ESM.pdf]

Extended Data

ZNF92, an unexplored transcription factor with remarkably distinct breast cancer over-expression associated with prognosis and cell-of-origin. Mohammad Kamran[1], Udayan Bhattacharya[1], Mohamed Omar[1], Luigi Marchionni [1] and Tan A. Ince[1,2] (1) Weill Cornell Medicine, Department of Pathology and Laboratory Medicine and (2) New York Presbyterian, Brooklyn Methodist Hospital

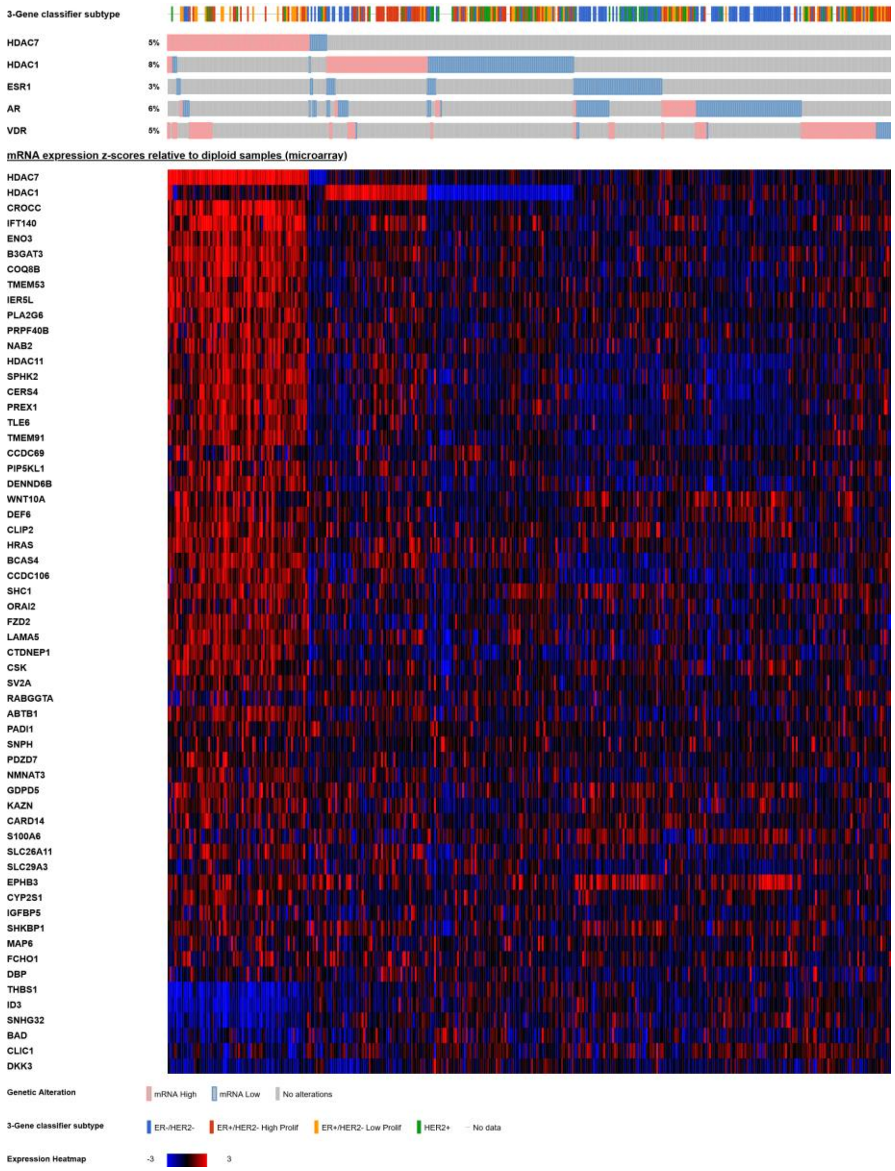

Supplementary Figure 1. HDAC1/7-SE upregulated genes in human breast cancer
The RNA Seq expression analysis of 125 genes upregulated by HDAC1/7-SE *in vitro* show that 63 of these genes correlate with HDAC7 expression *in vivo* (see Supplementary Table 1 for more details). The heatmap illustrates that 88% of these genes (56/63) positively correlate with HDAC7 over-expression in human breast cancers. The remaining 7 genes have an inverse correlation with HDAC7 in vivo, diverging from the in vitro results. HDAC7 co-expression and heatmap generated using cBioPortal online tools analyzing METABRIC data set complete samples (n=1,904) [1,2], mRNA expression z-scores relative to diploid samples [3]. Red= high expression, blue = low expression.

			1	2	3	4	5	6	7	8	9	10	
	Entrez ID	Gene Symbol	ZNF92	NEUROGENESIS	PROLIFERATION	ADHESION	LOCOMOTION	SIGNALING	ADHESION	TAXIS	MIGRATION	ZNF768	Gene Description
1	9289	ADGRG1	X	X	X	X	X	X	X		X	X	adhesion G protein-coupled receptor G1
2	2049	EPHB3	X	X		X	X		X	X	X		EPH receptor B3
3	1523	CUX1	X	X									cut like homeobox 1
4	9124	PDLIM1	X			X	X				X		PDZ and LIM domain 1
5	5097	PCDH1	X			X		X					protocadherin 1
6	84929	FIBCD1	X			X						X	fibrinogen C domain containing 1
7	8398	PLA2G6	X				X	X		X		X	phospholipase A2 group VI
8	79816	TLE6	X					X					TLE family member 6
9	27122	DKK3	X					X					dickkopf WNT signaling pathway inhibitor 3
10	55315	SLC29A3	X									X	solute carrier family 29 member 3
11	23149	FCHO1	X									X	FCH and mu domain containing endocytic adaptor 1
12	3855	KRT7	X									X	keratin 7
13	6337	SCNN1A	X									X	sodium channel epithelial 1 subunit alpha
14	79934	COQ8B	X									X	coenzyme Q8B
15	81544	GDPD5	X									X	glycerophosphodiester phosphodiesterase domain containing 5
16	4016	LOXL1	X										lysyl oxidase like 1
17	3985	LIMK2	X										LIM domain kinase 2
18	56848	SPHK2	X										sphingosine kinase 2
19	9696	CROCC	X										ciliary rootlet coiled-coil, rootletin
20	92799	SHKBP1	X										SH3KBP1 binding protein 1
21	1891	ECH1	X										enoyl-CoA hydratase 1
22	55653	BCAS4	X										breast carcinoma amplified sequence 4
23	80325	ABTB1	X										ankyrin repeat and BTB domain containing 1
24	113451	AZIN2	X										antizyme inhibitor 2
25	114783	LMTK3	X										lemur tyrosine kinase 3
26	5875	RABGGTA	X										Rab geranylgeranyltransferase subunit alpha
27	641649	TMEM91	X										transmembrane protein 91
28	4430	MYO1B	X										myosin IB
29	79639	TMEM53	X										transmembrane protein 53
30	3569	IL6		X	X	X	X	X	X	X	X		interleukin 6
31	5578	PRKCA		X	X	X	X	X	X	X	X		protein kinase C alpha
32	6376	CX3CL1		X	X	X	X	X	X	X	X		C-X3-C motif chemokine ligand 1
33	2736	GLI2		X	X	X	X	X	X	X			GLI family zinc finger 2
34	7042	TGFB2		X	X	X	X		X	X	X		transforming growth factor beta 2
35	2064	ERBB2		X	X	X	X		X	X			erb-b2 receptor tyrosine kinase 2
36	6464	SHC1		X	X	X	X			X	X		SHC adaptor protein 1
37	146433	IL34		X	X		X			X	X		interleukin 34
38	6277	S100A6		X	X								S100 calcium binding protein A6
39	4035	LRP1		X		X	X	X	X	X	X		LDL receptor related protein 1
40	50488	MINK1		X		X	X	X	X		X	X	misshapen like kinase 1
41	3911	LAMA5		X		X	X		X	X	X		laminin subunit alpha 5
42	57580	PREX1		X		X	X		X	X	X		PI-3,4,5-trisphosphate dependent Rac exchange factor 1
43	91653	BOC		X		X	X			X			BOC cell adhesion associated, oncogene regulated
44	1445	CSK		X		X			X			X	C-terminal Src kinase
45	80326	WNT10A		X				X				X	Wnt family member 10A
46	9751	SNPH		X				X				X	syntaphilin
47	2535	FZD2		X				X					frizzled class receptor 2
48	7475	WNT6		X				X					Wnt family member 6
49	79885	HDAC11		X								X	histone deacetylase 11
50	3399	ID3		X									inhibitor of DNA binding 3, HLH protein
51	9742	IFT140		X									intraflagellar transport 140
52	4135	MAP6		X									microtubule associated protein 6
53	79955	PDZD7		X									PDZ domain containing 7
54	4665	NAB2		X									NGFI-A binding protein 2
55	7057	THBS1			X	X	X		X	X	X		thrombospondin 1
56	572	BAD			X	X		X	X				BCL2 associated agonist of cell death
57	3485	IGFBP2			X	X		X	X				insulin like growth factor binding protein 2
58	3265	HRAS			X		X	X		X	X		HRas proto-oncogene, GTPase
59	2149	F2R			X		X	X			X		coagulation factor II thrombin receptor
60	2246	FGF1			X		X			X	X		fibroblast growth factor 1
61	1512	CTSH			X		X				X		cathepsin H
62	3488	IGFBP5			X		X				X		insulin like growth factor binding protein 5
63	5916	RARG			X			X					retinoic acid receptor gamma
64	2355	FOSL2			X							X	FOS like 2, AP-1 transcription factor subunit
65	894	CCND2			X								cyclin D2 [Source:HGNC Symbol;Acc:HGNC:1583]
66	7433	VIPR1			X								vasoactive intestinal peptide receptor 1
67	1277	COL1A1				X	X	X	X		X		collagen type I alpha 1 chain
68	29984	RHOD				X	X		X		X		ras homolog family member D
69	4017	LOXL2				X	X				X		lysyl oxidase like 2
70	1289	COL5A1				X	X				X		collagen type V alpha 1 chain
71	1192	CLIC1				X							chloride intracellular channel 1
72	138429	PIP5KL1					X				X		phosphatidylinositol-4-phosphate 5-kinase like 1
73	8140	SLC7A5					X				X		solute carrier family 7 member 5
74	27092	CACNG4						X					calcium voltage-gated channel auxiliary subunit gamma 4
75	9900	SV2A						X					synaptic vesicle glycoprotein 2A
76	6330	SCN4B						X					sodium voltage-gated channel beta subunit 4
77	23399	CTDNEP1						X					CTD nuclear envelope phosphatase 1
78	83439	TCF7L1						X					transcription factor 7 like 1
79	10587	TXNRD2										X	thioredoxin reductase 2
80	4151	MB										X	myoglobin
81	7461	CLIP2										X	CAP-Gly domain containing linker protein 2
82	55245	UQCC1										X	ubiquinol-cytochrome c reductase complex assembly factor 1
83	8714	ABCC2										X	ATP binding cassette subfamily C member 3
84	151056	PLB1										X	phospholipase B1
85	80852	GRIP2										X	glutamate receptor interacting protein 2
86	284129	SLC26A11										X	solute carrier family 26 member 11

Supplementary Figure 2. The top ten gene sets (columns) associated with HDAC1/7-SE upregulated genes (rows) in gene get enrichment analysis (GSEA). See Table S3c for details. The analysis is carried out using the GSEA online tool.

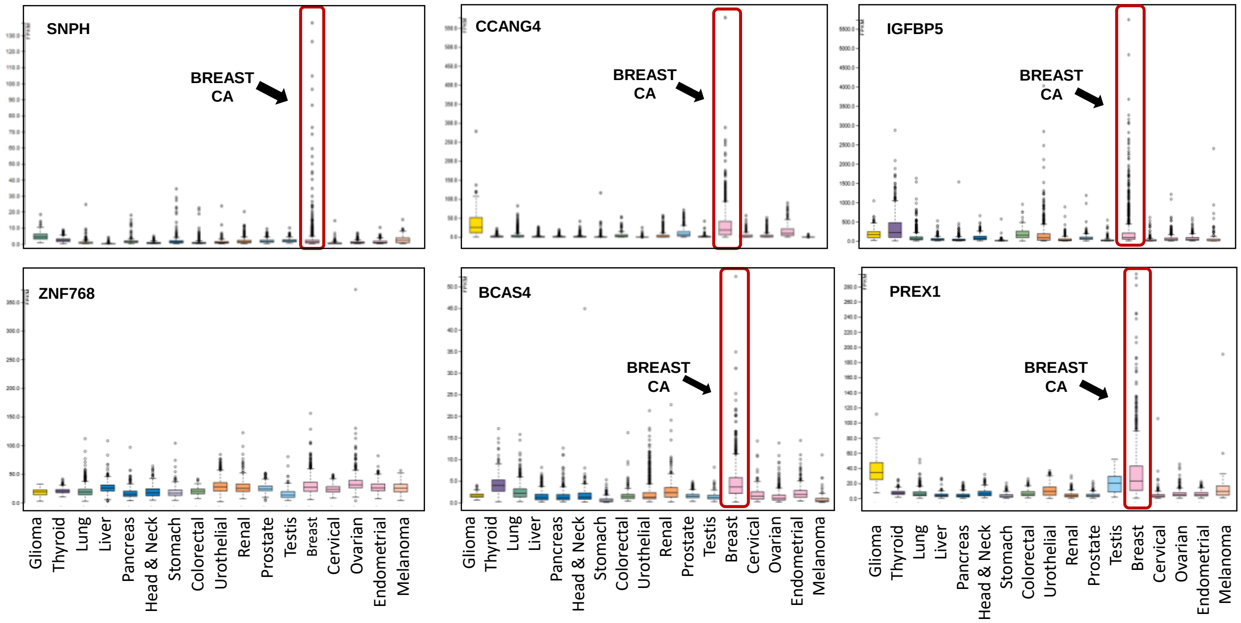

Supplementary Figure 3. Breast cancer specific expression of HDCA1/7 downstream targets

Human Protein Atlas (HPA) PanCancer expression analysis of **SNPH** (*Synaptaphillin*) **CACNG4** (Calcium voltage-gated channel auxiliary subunit gamma 4), **IGFBP5** (*insulin like growth factor binding protein 5*), **ZNF768** (*Zinc Finger Protein 768*), **BCAS4** (*breast carcinoma amplified sequence 4*), **PREX1** (*phosphatidylinositol-3,4,5-trisphosphate dependent Rac exchange factor 1*). The RNA-seq data from 17 cancer types is visualized with box plots, shown as median and 25th and 75th percentiles. Points are displayed as outliers if they are above or below 1.5 times the interquartile range.

A.

	ET-60	ZNF92 Target	in vivo HDAC7 correlation	Survival correlation
1	GDPD5	ZNF92	HDAC7	Yes
2	ABTB1	ZNF92	HDAC7	Yes
3	FCHO1	ZNF92	HDAC7	Yes
4	PLA2G6	ZNF92	HDAC7	Yes
5	SHKBP1	ZNF92	HDAC7	Yes
6	BCAS4	ZNF92	HDAC7	
7	EPHB3	ZNF92	HDAC7	
8	CROCC	ZNF92	HDAC7	
9	RABGGTA	ZNF92	HDAC7	
10	TMEM53	ZNF92	HDAC7	
11	FIBCD1	ZNF92		Yes
12	DKK3	ZNF92		Yes
13	ECH1	ZNF92		Yes
14	LIMK2	ZNF92		Yes
15	PCDH1	ZNF92		Yes
16	SCNN1A	ZNF92		Yes
17	ADGRG1	ZNF92		
18	CUX1	ZNF92		
19	LOXL1	ZNF92		
20	KRT7	ZNF92		
21	MYO1B	ZNF92		
22	PDLIM1	ZNF92		
23	CCDC69		HDAC7	Yes
24	IGFBP5		HDAC7	Yes
25	MAP6		HDAC7	Yes
26	CYP2S1		HDAC7	Yes
27	S100A6		HDAC7	Yes
28	WNT10A		HDAC7	Yes
29	CSK		HDAC7	Yes
30	DEF6		HDAC7	Yes

		ZNF92 Target	in vivo HDAC7 correlation	Survival correlation
31	IER5L		HDAC7	Yes
32	PRPF40B		HDAC7	Yes
33	HDAC11		HDAC7	
34	PREX1		HDAC7	
35	DENND6B		HDAC7	
36	ENO3		HDAC7	
37	FZD2		HDAC7	
38	CTDNEP1		HDAC7	
39	LAMA5		HDAC7	
40	NAB2		HDAC7	
41	SHC1		HDAC7	
42	SNPH		HDAC7	
43	CACNG4			Yes
44	CX3CL1			Yes
45	SUSD2			Yes
46	BNIPL			Yes
47	BOC			Yes
48	ID3			Yes
49	LRP1			Yes
50	MGAT1			Yes
51	THBS1			Yes
52	CCND2			Yes
53	CPA4			Yes
54	LOXL2			Yes
55	FGF1			Yes
56	IL6			Yes
57	MB			Yes
58	VIPR1			Yes
59	XPC			Yes
60	TENT5B			Yes

B.

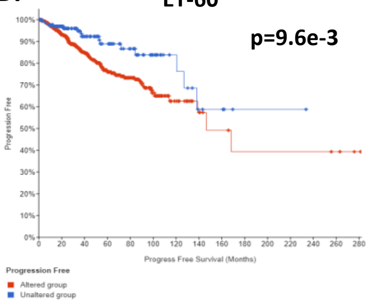

C.

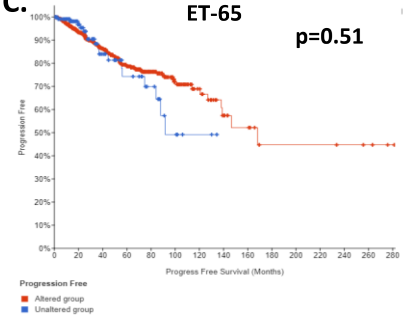

Supplementary Figure 4. ET-60 signature

A) The list of ET-60 genes that have a ZNF92 binding site in their promoter (± 1,000 bp), correlate with HDAC7 expression in vivo (see Supplementary Fig. 1 and Supplementary Table 2), or have been associated with breast cancer survival. **B-C)** While the ET-60 signature significantly correlates with progression free survival in TCGA breast cancer dataset (p=9.3e-3, Log Rank test, n=1,082 PanCancer, cBioPortal), the remaining 65 genes that make up the ET-125 signature are non-significant (p=0.51, Log Rank test)

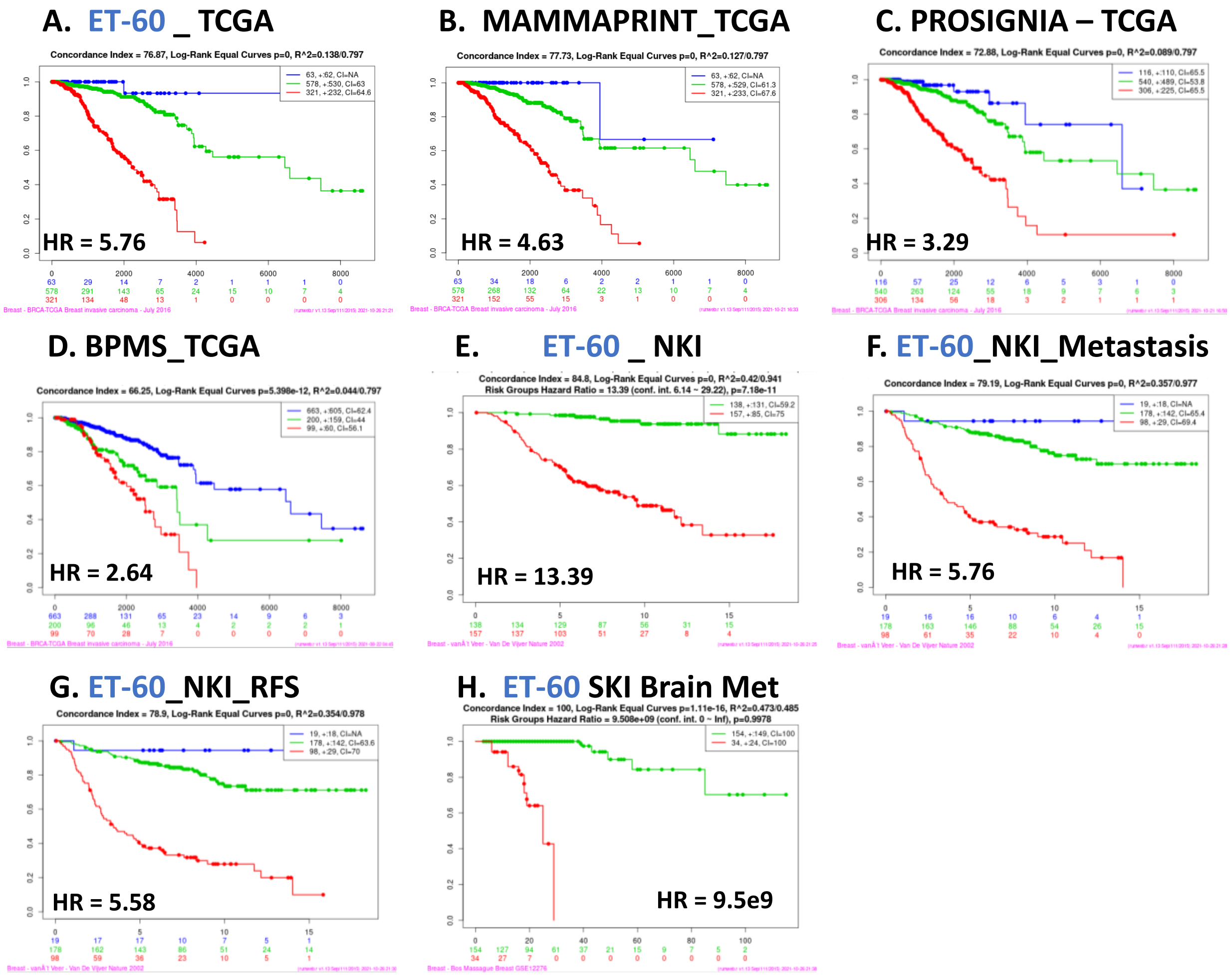

Supplementary Figure 5. ET-60 prognostic groups compared to other signatures

The Kaplan-Meier (KM) survival charts of human breast cancer in the BRCA_TCGA 2016 dataset (a-d), NKI dataset (e-g) and SKI GSE12276 data set (h) generated using SurvExpress; high risk (red line), medium risk (green line), low risk (blue line) groups determined by auto-selection of cutoff values and maximized risk groups[4]. See Supplementary Fig. 6 for equal risk groups (tertiles). **A)** KM survival chart of ET-60 expression in TCGA, HR: 5.76 (CI: 4.0 – 8.2); **B)** KM survival chart of 70-gene signature in TCGA (Mammaprint); HR: 4.63 (CI: 3.3 - 6.6); four genes were not found in TCGA Breast invasive carcinoma - July 2016 dataset AA555029_RC, LOC100131053, LOC100288906, LOC730018; **C)** KM survival chart of 50-gene signature in TCGA (PAM50/Prosignia), HR: 3.29 (CI: 2.4 - 4.4); all genes found in the dataset; **D)** KM survival chart of 25-gene signature (BPMS) in TCGA, HR: 2.64 (CI: 2.0 - 3.4). 3 Genes not found in the dataset: ZH3H3, HS3STSB1, PDEC1; **E)** Survival KM chart of ET-60 expression in the NKI dataset, HR: 13.39 (CI: 6.1 – 29.2); **F)** Time to metastasis KM chart of ET-60 expression in the NKI dataset, HR: 5.76 (CI: 3.8 – 8.5); **G)** Time to recurrence KM chart of ET-60 expression in the NKI dataset, HR: 5.58 (CI: 3.7 – 8.2); **H)** Time to brain relapse KM chart of ET-60 expression in the SKI dataset, HR: 9.5×10^9. The relative hazard ratios (HR) were computed using Cox proportional hazard regression analysis.

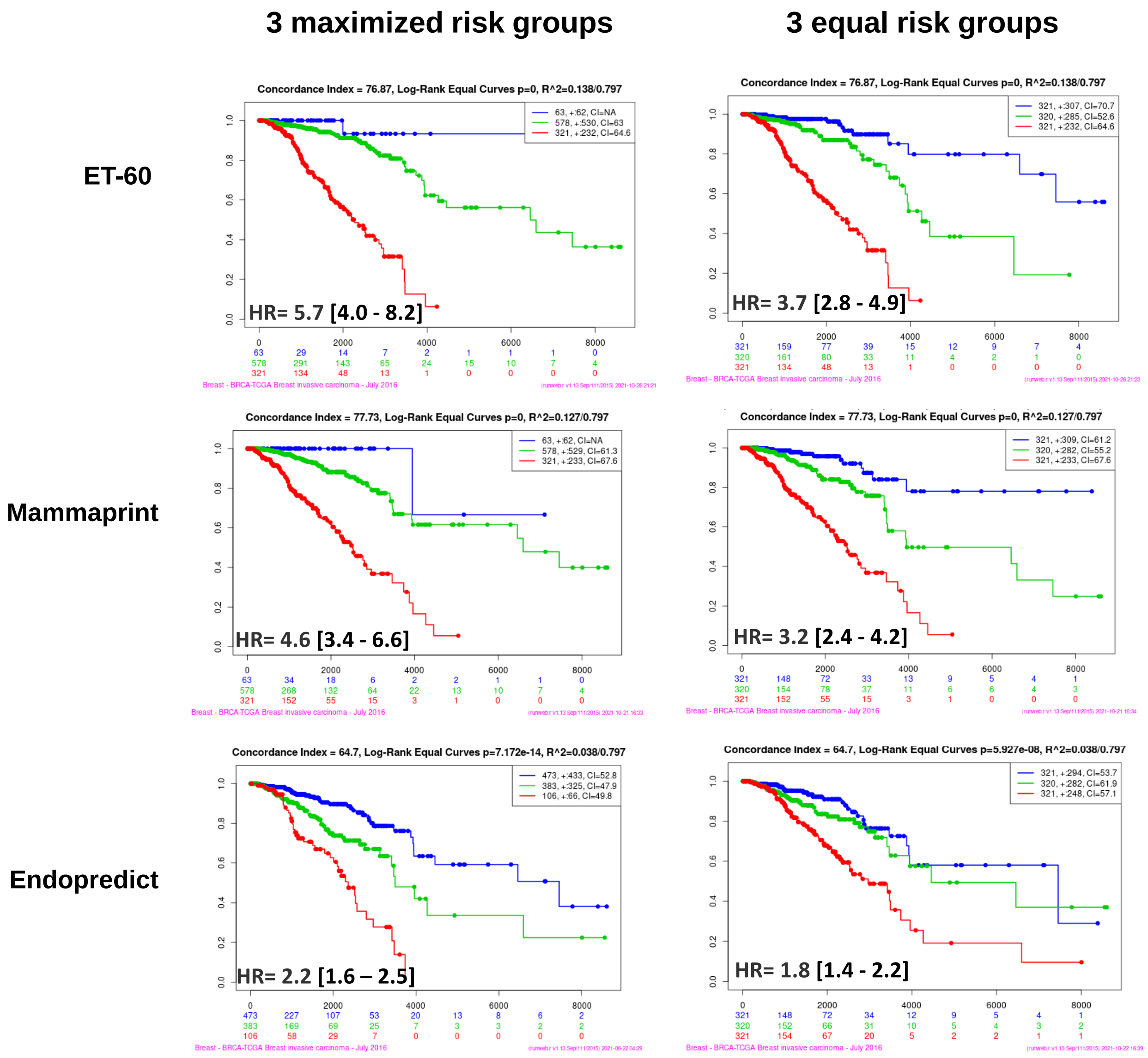

Supplementary Figure 6. ET-60 tertiles vs. maximized risk groups

The risk groups in SurvExpress are generated by two methods[4]. One method generates risk groups with equal number of samples by splitting the ordered prognostic index (PI), where higher values reflect higher risk. For two risk groups, this would be the median cut-off, for three groups it would be tertiles (three groups with equal sample numbers). The second method determines the risk groups through a log-rank test that is performed along all values of the ordered PI and an algorithm chooses the cut-off point where the p-value is minimum. We observed similar relative results with both approaches for ET-60, Mammaprint and Endopredict. The relative hazard ratios (HR) were computed using Cox proportional hazard regression analysis

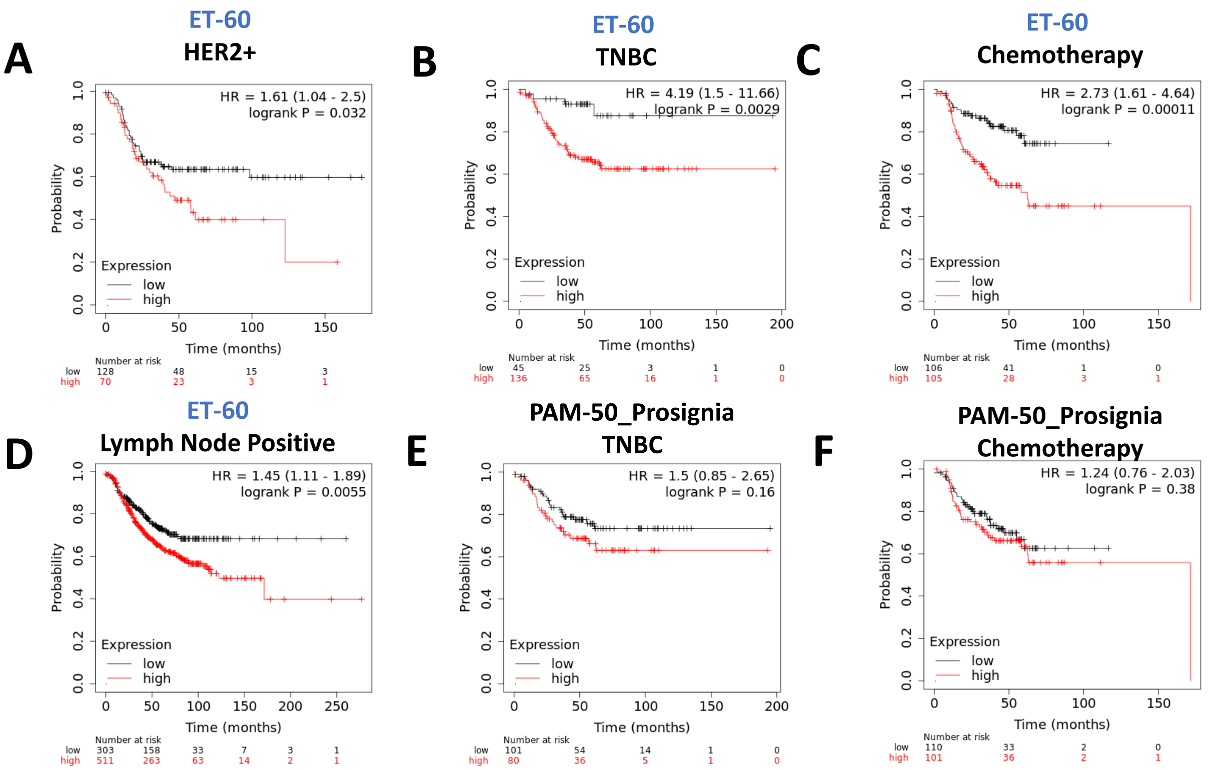

Supplementary Figure 7. ET-60 in breast cancer subgroups

The Kaplan-Meier (KM) charts of relapse free survival of human breast cancer generated using Kaplan-Meier plotter [Breast] high risk (red line), low risk (black line). The analysis is carried out with user selected probe sets with auto selection for best cut off, exclusion of biased arrays, and no data censoring and multivariate analysis. The relative hazard ratios (HR) were computed using Cox proportional hazard regression analysis [5] .

A) KM chart of ET-60 in HER2+ human breast cancer, HR: 1.61 [CI 1.04-2.5], p=0.032.

B) KM chart of ET-60 in triple negative breast cancer (TNBC), HR: 4.19 [CI 1.5-11.66], p=0.0029.

C) KM chart of ET-60 in breast cancer patients with systemic chemotherapy, HR: 2.73 [CI 1.61-4.64], p=0.00011.

D) KM chart of ET-60 in lymph node positive human breast cancer, HR: 1.45 [CI 1.11-1.89], p=0.0055.

E) KM chart of PAM50 (Prosignia) in triple negative breast cancer (TNBC), HR: 1.5 [CI 0.85-2.65], p=0.16.

F) KM chart of PAM50 (Prosignia) in breast cancer patients with systemic chemotherapy, HR: 1.24 [CI 0.76-2.03], p=0.38.

A.

	Gene Name	Selection	ET-9
1	FIBCD1	SGR	+
2	MAP6	SGR	+
3	CACNG4	SGR	+
4	GDPD5	k-TSPs	+
5	CCDC69	k-TSPs	+
6	CX3CL1	k-TSPs	+
7	IGFBP5	k-TSPs	+
8	SUSD2	SGS	+
9	ADGRG1	SGS	+
10	ID3	k-TSPs	-
11	CUX1	k-TSPs	-
12	IER5L	k-TSPs	-
13	S100A6	SGR	-
14	SHC1	SGR	-
15	MB	SGS	-
16	ACSF2	SGS	-
17	ABCC2	SGS	-

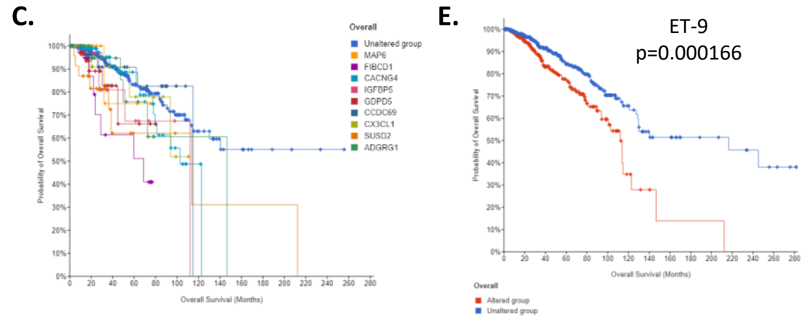

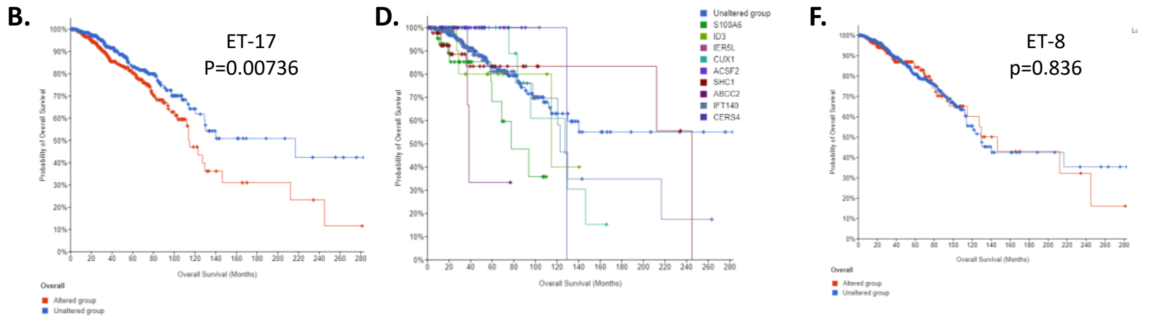

Supplementary Figure 8. ET-9 signature

A) We carried out k-top scoring pairs (k-TSPs) [6,7], leave-one-out single gene removal (SGR) and single gene significance (SGS) analysis on the 125 genes in the ET-125 signature, which identified 17 genes (ET-17) in Metabric and TCGA breast cancer datasets.

B) The ET-17 signature has a significant association with progression free survival in the TCGA breast cancer dataset (p=0.00736, Log Rank test).

C-D) Each of the genes in the ET-17 signature were analyzed individually, which was used to divide this signature into two subsets ET-9 (c) and ET8 (d).

E) The ET-9 signature maintained the prognostic power of ET-17 (p= 0.0001, Log Rank test. TCGA breast cancer dataset).

F) The ET-8 signature was not prognostic (p= 0.8, Log Rank test, TCGA breast cancer dataset).

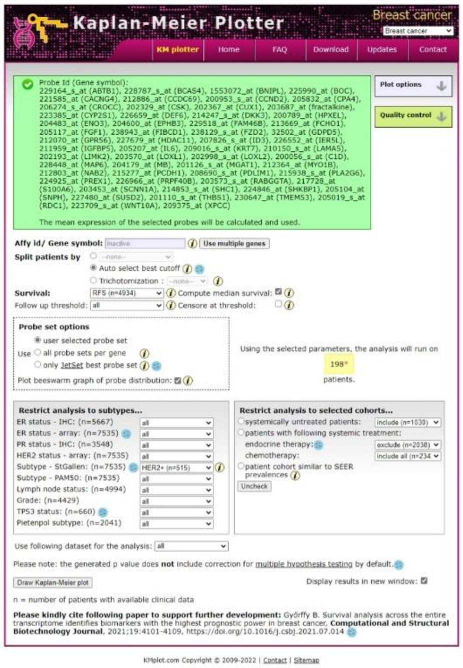

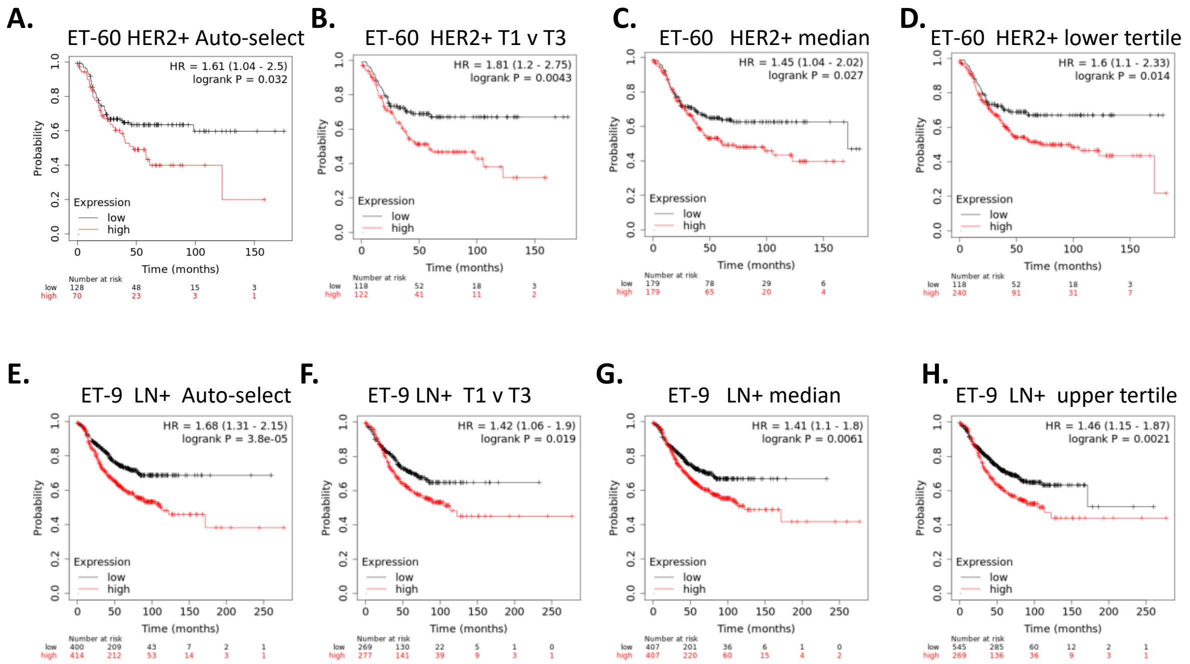

Supplementary Figure 9. Comparison of cut-off methods

The KM plotter platform provides various methods to split the patient population into risk groups including auto select best cutoff that we used throughout this study. We also observed similar results with Trichotomization (T1 vs. T3), median and lower tertile cut-offs. An example is shown for HER2+ breast cancers using ET-60 signature (A-D) and with lymph node positive breast cancers with ET-9 signature (E-H). The relative hazard ratios (HR) were computed using Cox proportional hazard regression analysis.

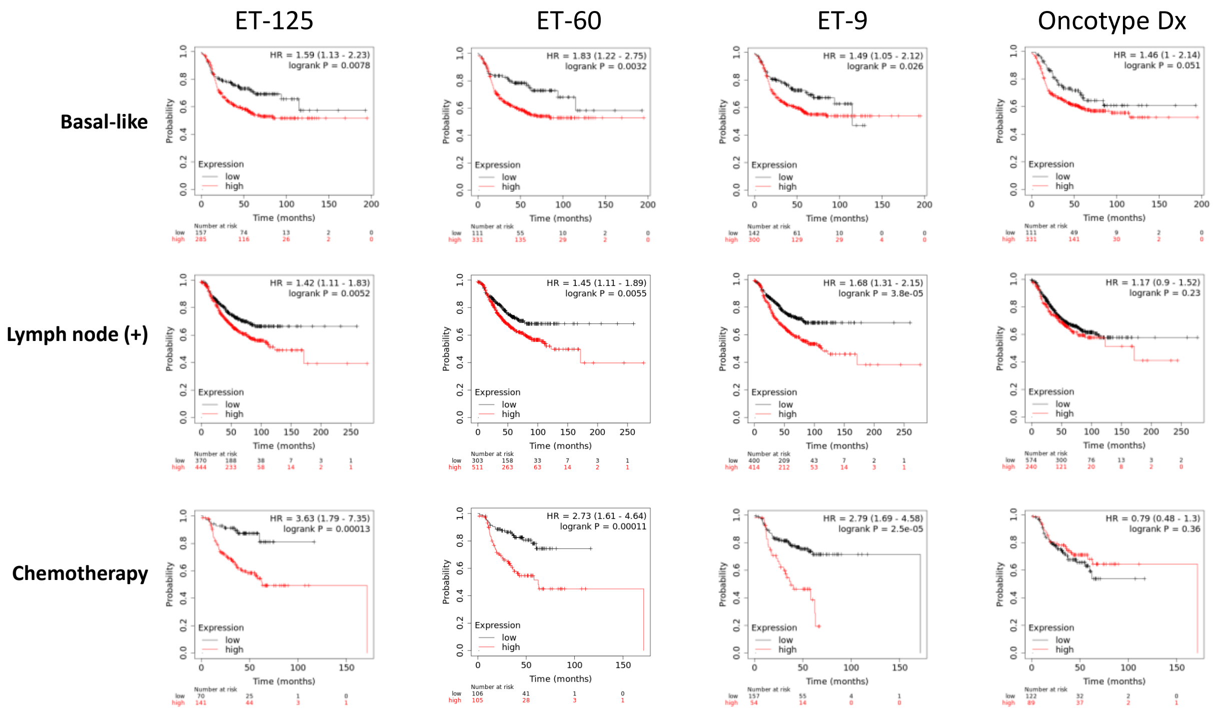

Supplementary Figure 10. Comparison of ET-125, ET-60, ET-9 and Oncotype Dx

While the nine gene ET-9 signature is similarly prognostic as ET-60 or ET-15 in basal-like, lymph node-positive and chemotherapy treated breast cancers, the 21 gene Oncotype Dx is not significantly prognostic. The Kaplan-Meier (KM) charts of relapse free survival of human breast cancer generated using Kaplan-Meier plotter [Breast] high risk (red line), low risk (black line). The analysis is carried out with user selected probe sets with auto selection for best cut off, exclusion of biased arrays, and multivariate analysis[5]. The relative hazard ratios (HR) were computed using Cox proportional hazard regression analysis.

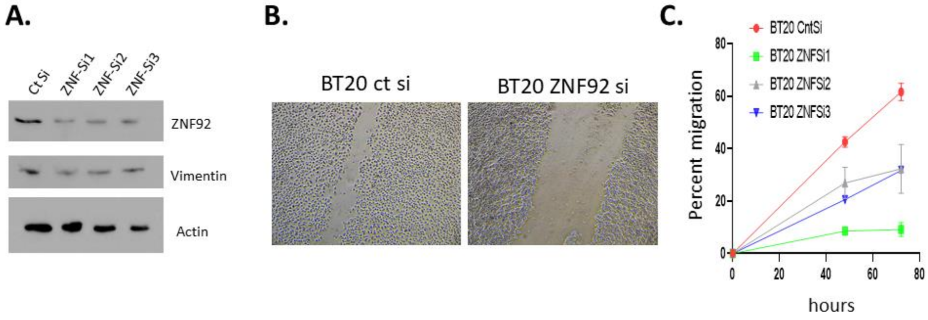

Supplementary Figure 11. ZNF92 knock-down

A) ZNF92 si1, si2 and si3 (Ambion-AM16708- ID238043, 110198, 110199) were used for knock-down of ZNF92 in BT20 cells. Western blot shows that all three siRNAs resulted in down-regulation of ZNF92 protein. In GSEA analysis the ET-125 signature was associated with epithelial-mesenchymal transition (EMT). Consistent with that, ZNF92 inhibition also down-regulated vimentin expression. See supplementary figure 15 for the uncropped scans of the entire film for the western blot.

B) Micrographs show the scratch assay. While the control cells migrated to fill out the gap the ZNF92 knock-down cells failed migrate as well.

C) Chart shows the migration of cells quantified by ImageJ area tool.

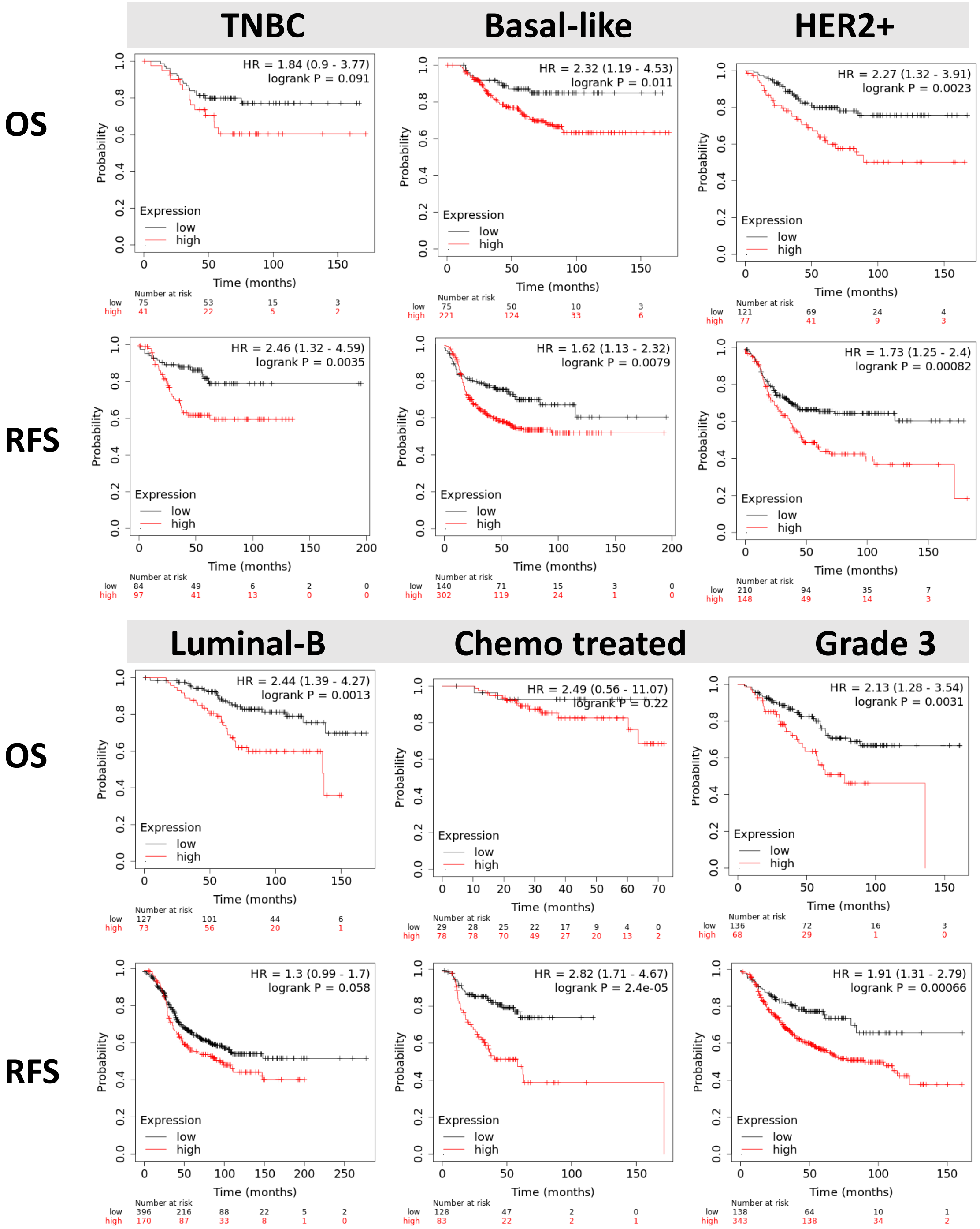

Supplementary Figure 12. ZNF92 target genes

The 29 genes that have ZNF92 binding sites in their promoter were tested in triple-negative carcinoma (TNBC), basal-like, HER2+, Luminal-B, chemotherapy treated and grade 3 breast cancers. Kaplan-Meier plotter [Breast] high risk (red line), low risk (black line). The analysis is carried out with user selected probe sets with auto selection for best cut off, exclusion of biased arrays, and multivariate analysis [5], OS = overall survival, RFS = relapse free survival. The relative hazard ratios (HR) were computed using Cox proportional hazard regression analysis.

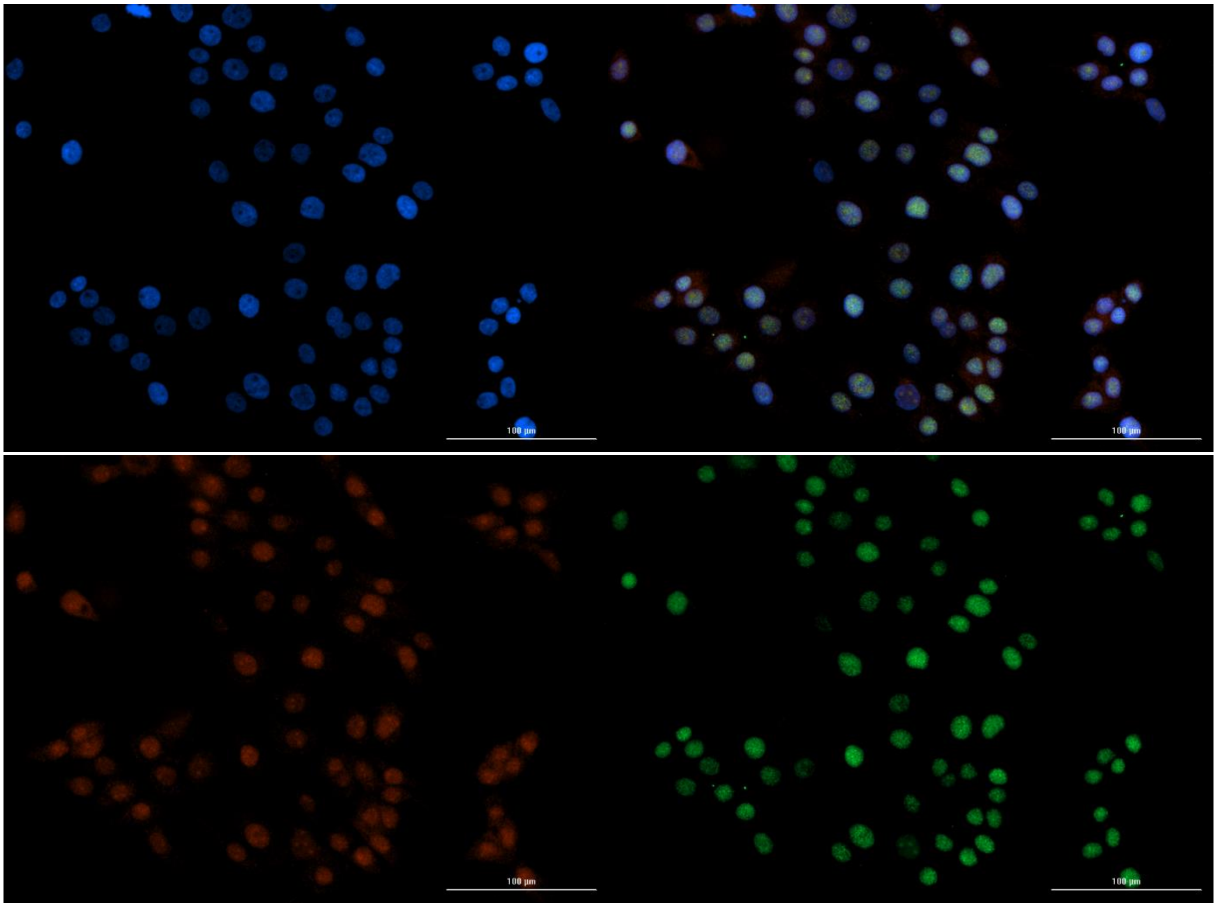

Supplementary Figure 13. Uncropped and unprocessed immunofluorescence image

The images correspond to figure 6 b; DAPI (upper left), HDAC7 (lower left), ZNF92 (lower right), merged image of DAPI, HDAC7 and ZNF92 (upper right).

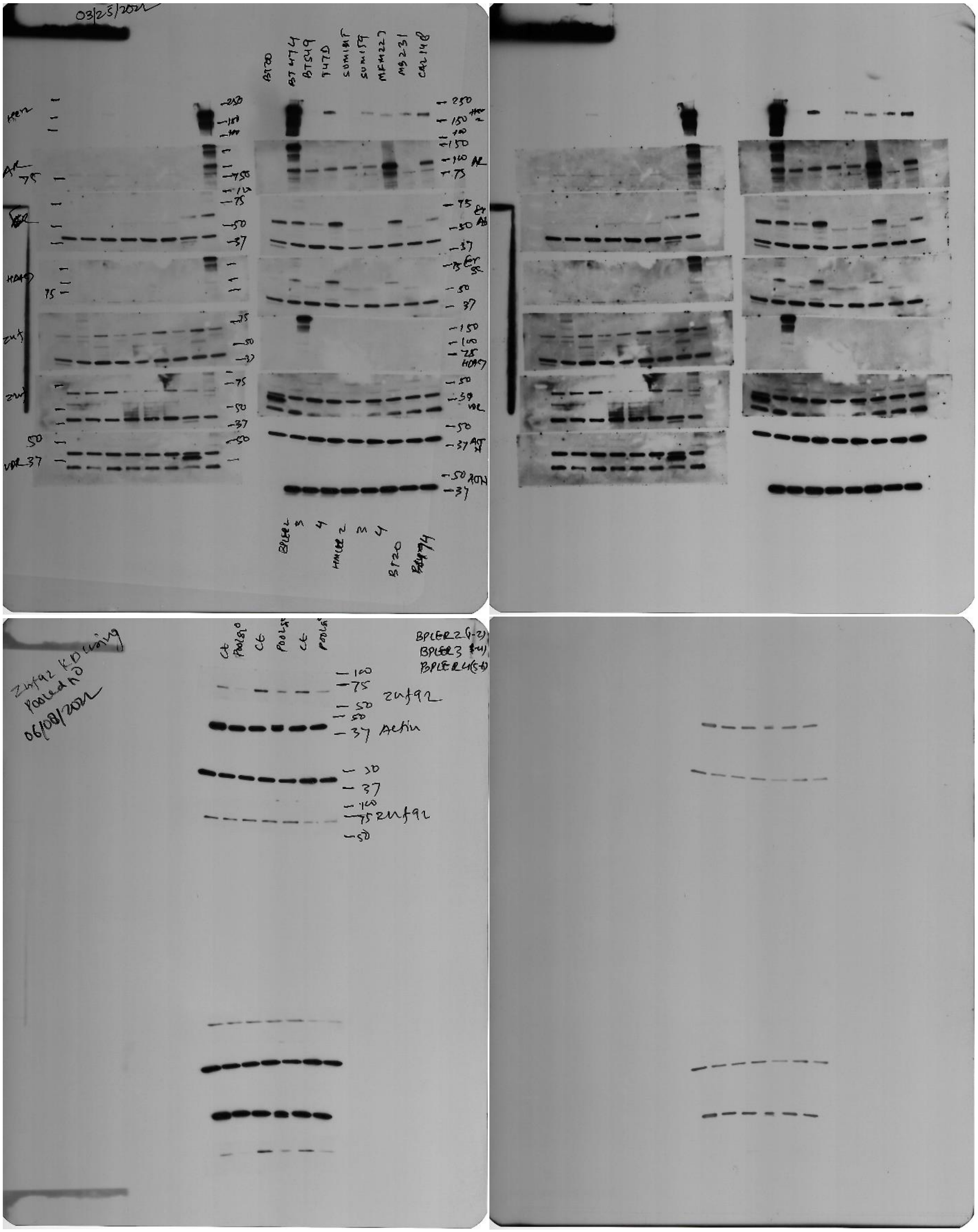

Supplementary Figure 14. Uncropped and unprocessed gel image

The uncropped scans of the film for the western blots in figure 6 c-d.

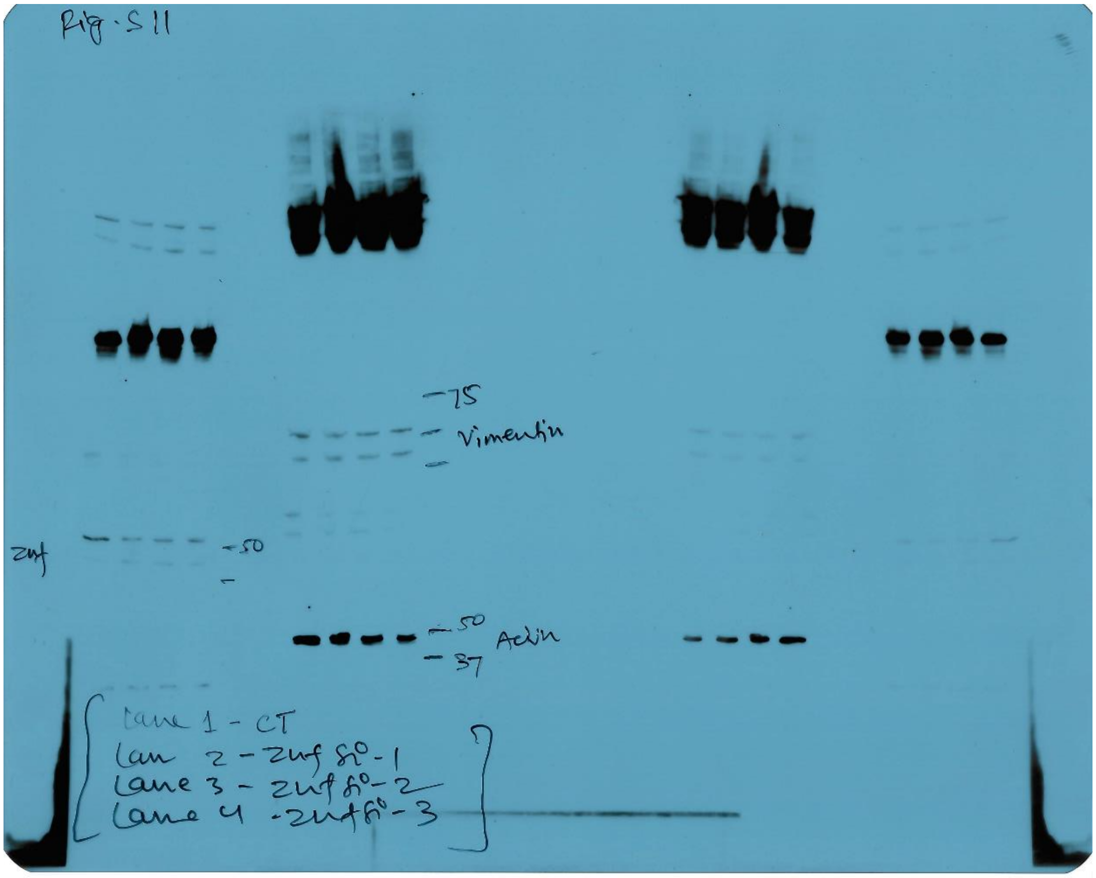

Supplementary Figure 15. Uncropped gel image

The uncropped scan of the entire film for the western blot in supplementary figure 11.

	Entrez	Symbol	Gene Description
1	9289	ADGRG1	adhesion G protein-coupled receptor G1
2	2049	EPHB3	EPH receptor B3
3	1523	CUX1	cut like homeobox 1
4	9124	PDLIM1	PDZ and LIM domain 1
5	5097	PCDH1	protocadherin 1
6	84929	FIBCD1	fibrinogen C domain containing 1
7	8398	PLA2G6	phospholipase A2 group VI
8	79816	TLE6	"TLE family member 6, subcortical maternal complex
9	27122	DKK3	dickkopf WNT signaling pathway inhibitor 3
10	55315	SLC29A3	solute carrier family 29 member 3
11	23149	FCHO1	FCH and mu domain containing endocytic adaptor 1
12	3855	KRT7	keratin 7
13	6337	SCNN1A	sodium channel epithelial 1 subunit alpha
14	79934	COQ8B	coenzyme Q8B
15	81544	GDPD5	glycerophosphodiester phosphodiesterase domain
16	4016	LOXL1	lysyl oxidase like 1
17	3985	LIMK2	LIM domain kinase 2
18	56848	SPHK2	sphingosine kinase 2
19	9696	CROCC	"ciliary rootlet coiled-coil, rootletin
20	92799	SHKBP1	SH3KBP1 binding protein 1
21	1891	ECH1	enoyl-CoA hydratase 1
22	55653	BCAS4	breast carcinoma amplified sequence 4
23	80325	ABTB1	ankyrin repeat and BTB domain containing 1
24	113451	AZIN2	antizyme inhibitor 2
25	114783	LMTK3	lemur tyrosine kinase 3
26	5875	RABGGTA	Rab geranylgeranyltransferase subunit alpha
27	641649	TMEM91	transmembrane protein 91
28	4430	MYO1B	myosin IB
29	79639	TMEM53	transmembrane protein 53
30	3569	IL6	interleukin 6
31	5578	PRKCA	protein kinase C alpha
32	6376	CX3CL1	C-X3-C motif chemokine ligand 1
33	2736	GLI2	GLI family zinc finger 2
34	7042	TGFB2	transforming growth factor beta 2
35	2064	ERBB2	erb-b2 receptor tyrosine kinase 2
36	6464	SHC1	SHC adaptor protein 1
37	146433	IL34	interleukin 34
38	6277	S100A6	S100 calcium binding protein A6
39	4035	LRP1	LDL receptor related protein 1
40	50488	MINK1	misshapen like kinase 1
41	3911	LAMA5	laminin subunit alpha 5
42	57580	PREX1	PI-3,4,5-trisphosphate dependent Rac exchange factor 1
43	91653	BOC	"BOC cell adhesion associated, oncogene regulated
44	1445	CSK	C-terminal Src kinase
45	80326	WNT10A	Wnt family member 10A
46	9751	SNPH	syntaphilin
47	2535	FZD2	frizzled class receptor 2
48	7475	WNT6	Wnt family member 6
49	79885	HDAC11	histone deacetylase 11
50	3399	ID3	"inhibitor of DNA binding 3, HLH protein
51	9742	IFT140	intraflagellar transport 140
52	4135	MAP6	microtubule associated protein 6
53	79955	PDZD7	PDZ domain containing 7
54	4665	NAB2	NGFI-A binding protein 2
55	7057	THBS1	thrombospondin 1
56	572	BAD	BCL2 associated agonist of cell death
57	3485	IGFBP2	insulin like growth factor binding protein 2
58	3265	HRAS	"HRas proto-oncogene, GTPase
59	2149	F2R	coagulation factor II thrombin receptor
60	2246	FGF1	fibroblast growth factor 1
61	1512	CTSH	cathepsin H
62	3488	IGFBP5	insulin like growth factor binding protein 5
63	5916	RARG	retinoic acid receptor gamma
64	2355	FOSL2	"FOS like 2, AP-1 transcription factor subunit
65	894	CCND2	cyclin D2
66	7433	VIPR1	vasoactive intestinal peptide receptor 1
67	1277	COL1A1	collagen type I alpha 1 chain
68	29984	RHOD	ras homolog family member D
69	4017	LOXL2	lysyl oxidase like 2
70	1289	COL5A1	collagen type V alpha 1 chain
71	1192	CLIC1	chloride intracellular channel 1
72	138429	PIP5KL1	phosphatidylinositol-4-phosphate 5-kinase like 1
73	8140	SLC7A5	solute carrier family 7 member 5
74	27092	CACNG4	calcium voltage-gated channel auxiliary subunit gamma 4
75	9900	SV2A	synaptic vesicle glycoprotein 2A
76	6330	SCN4B	sodium voltage-gated channel beta subunit 4
77	23399	CTDNEP1	CTD nuclear envelope phosphatase 1
78	83439	TCF7L1	transcription factor 7 like 1
79	10587	TXNRD2	thioredoxin reductase 2
80	4151	MB	myoglobin
81	7461	CLIP2	CAP-Gly domain containing linker protein 2
82	55245	UQCC1	ubiquinol-cytochrome c reductase complex assembly factor 1
83	8714	ABCC3	ATP binding cassette subfamily C member 3
84	151056	PLB1	phospholipase B1
85	80852	GRIP2	glutamate receptor interacting protein 2
86	284129	SLC26A11	solute carrier family 26 member 11
87	149428	BNIPL	BCL2 interacting protein like
88	284086	NEK8	NIMA related kinase 8
89	10826	FAXDC2	fatty acid hydroxylase domain containing 2
90	79092	CARD14	caspase recruitment domain family member 14
91	1244	ABCC2	ATP binding cassette subfamily C member 2
92	26229	B3GAT3	"beta-1,3-glucuronyltransferase 3
93	286	ANK1	ankyrin 1
94	192683	SCAMP5	secretory carrier membrane protein 5
95	79603	CERS4	ceramide synthase 4
96	3852	KRT5	keratin 5
97	23254	KAZN	"kazrin, periplakin interacting protein
98	144501	KRT80	keratin 80
99	10602	CDC42EP3	CDC42 effector protein 3
100	29903	CCDC106	coiled-coil domain containing 106
101	2027	ENO3	enolase 3
102	50854	SNHG32	small nucleolar RNA host gene 32
103	1628	DBP	D-box binding PAR bZIP transcription factor
104	389792	IER5L	immediate early response 5 like
105	64342	HS1BP3	HCLS1 binding protein 3
106	4245	MGAT1	alpha-1,3-mannosyl-glycoprotein 2-beta-N-
107	25766	PRPF40B	pre-mRNA processing factor 40 homolog B
108	50619	DEF6	DEF6 guanine nucleotide exchange factor
109	414918	DENND6B	DENN domain containing 6B
110	80221	ACSF2	acyl-CoA synthetase family member 2
111	149076	ZNF362	zinc finger protein 362
112	115572	TENT5B	terminal nucleotidyltransferase 5B
113	29943	PADI1	peptidyl arginine deiminase 1
114	1E+08	RNU6ATAC	"RNA, U6atac small nuclear (U12-dependent splicing)
115	51200	CPA4	carboxypeptidase A4
116	7508	XPC	"XPC complex subunit, DNA damage recognition and repair
117	29785	CYP2S1	cytochrome P450 family 2 subfamily S member 1
118	10867	TSPAN9	tetraspanin 9
119	80228	ORAI2	ORAI calcium release-activated calcium modulator 2
120	26112	CCDC69	coiled-coil domain containing 69
121	155368	METTL27	methyltransferase like 27
122	56241	SUSD2	sushi domain containing 2
123	349565	NMNAT3	nicotinamide nucleotide adenylyltransferase 3
124	340385	ZNF517	zinc finger protein 517
125	84303	CHCHD6	coiled-coil-helix-coiled-coil-helix domain containing 6

Supplementary Table 1.
The list of 125 named genes associated by super-enhancers and upregulated both by HDAC1 and HDAC7 in BPLER cells.

	A	B	Neither	A Not B	B Not A	Both	Log2 Odds Ratio	p-Value	q-Value	Tendency
1	HDAC7	CROCC	1776	43	38	47	>3	<0.001	<0.001	Co-occurrence
4	HDAC7	IFT140	1633	41	181	49	>3	<0.001	<0.001	Co-occurrence
5	HDAC7	ENO3	1777	61	37	29	>3	<0.001	<0.001	Co-occurrence
6	HDAC7	B3GAT3	1753	57	61	33	>3	<0.001	<0.001	Co-occurrence
7	HDAC7	COQ8B	1753	59	61	31	>3	<0.001	<0.001	Co-occurrence
8	HDAC7	TMEM53	1762	61	52	29	>3	<0.001	<0.001	Co-occurrence
9	HDAC7	IER5L	1761	61	53	29	>3	<0.001	<0.001	Co-occurrence
10	HDAC7	PLA2G6	1769	65	45	25	>3	<0.001	<0.001	Co-occurrence
12	HDAC7	PRPF40B	1749	66	65	24	>3	<0.001	<0.001	Co-occurrence
13	HDAC7	NAB2	1724	64	90	26	2.96	<0.001	<0.001	Co-occurrence
14	HDAC7	HDAC11	1748	68	66	22	>3	<0.001	<0.001	Co-occurrence
15	HDAC7	SPHK2	1717	66	97	24	2.686	<0.001	<0.001	Co-occurrence
16	HDAC7	CERS4	1740	69	74	21	2.839	<0.001	<0.001	Co-occurrence
17	HDAC7	PREX1	1722	67	92	23	2.684	<0.001	<0.001	Co-occurrence
18	HDAC7	TLE6	1752	71	62	19	2.919	<0.001	<0.001	Co-occurrence
19	HDAC7	TMEM91	1734	69	80	21	2.722	<0.001	<0.001	Co-occurrence
21	HDAC7	CCDC69	1752	73	62	17	2.718	<0.001	<0.001	Co-occurrence
22	HDAC7	PIP5KL1	1742	72	72	18	2.597	<0.001	<0.001	Co-occurrence
23	HDAC7	DENND6B	1720	70	94	20	2.386	<0.001	<0.001	Co-occurrence
24	HDAC7	WNT10A	1748	73	66	17	2.625	<0.001	<0.001	Co-occurrence
25	HDAC7	DEF6	1746	73	68	17	2.58	<0.001	<0.001	Co-occurrence
26	HDAC7	CLIP2	1742	73	72	17	2.494	<0.001	<0.001	Co-occurrence
27	HDAC7	HRAS	1724	72	90	18	2.26	<0.001	<0.001	Co-occurrence
28	HDAC7	BCAS4	1683	69	131	21	1.967	<0.001	<0.001	Co-occurrence
29	HDAC7	CCDC106	1683	69	131	21	1.967	<0.001	<0.001	Co-occurrence
30	HDAC7	SHC1	1694	71	120	19	1.918	<0.001	<0.001	Co-occurrence
31	HDAC7	ORAI2	1737	75	77	15	2.174	<0.001	<0.001	Co-occurrence
32	HDAC7	FZD2	1739	76	75	14	2.095	<0.001	<0.001	Co-occurrence
33	HDAC7	LAMA5	1698	73	116	17	1.769	<0.001	0.001	Co-occurrence
34	HDAC7	CTDNEP1	1660	70	154	20	1.623	<0.001	0.001	Co-occurrence
36	HDAC7	CSK	1716	75	98	15	1.808	<0.001	0.002	Co-occurrence
37	HDAC7	SV2A	1748	78	66	12	2.027	<0.001	0.002	Co-occurrence
38	HDAC7	RABGGTA	1721	76	93	14	1.769	<0.001	0.003	Co-occurrence
39	HDAC7	ABTB1	1719	76	95	14	1.737	<0.001	0.004	Co-occurrence
40	HDAC7	PADI1	1741	78	73	12	1.875	<0.001	0.004	Co-occurrence
42	HDAC7	SNPH	1761	80	53	10	2.054	<0.001	0.005	Co-occurrence
43	HDAC7	PDZD7	1760	80	54	10	2.026	<0.001	0.005	Co-occurrence
44	HDAC7	NMNAT3	1759	80	55	10	1.999	<0.001	0.006	Co-occurrence
45	HDAC7	GDPD5	1724	77	90	13	1.693	<0.001	0.007	Co-occurrence
46	HDAC7	KAZN	1717	77	97	13	1.579	0.001	0.011	Co-occurrence
47	HDAC7	CARD14	1713	77	101	13	1.518	0.002	0.014	Co-occurrence
48	HDAC7	S100A6	1725	78	89	12	1.576	0.002	0.015	Co-occurrence
49	HDAC7	SLC26A11	1672	74	142	16	1.348	0.002	0.015	Co-occurrence
50	HDAC7	SLC29A3	1735	79	79	11	1.613	0.003	0.017	Co-occurrence
51	HDAC7	EPHB3	1683	75	131	15	1.361	0.003	0.017	Co-occurrence
52	HDAC7	CYP2S1	1746	80	68	10	1.682	0.003	0.018	Co-occurrence
53	HDAC7	IGFBP5	1717	78	97	12	1.445	0.004	0.024	Co-occurrence
54	HDAC7	SHKBP1	1710	78	104	12	1.339	0.007	0.035	Co-occurrence
55	HDAC7	MAP6	1736	80	78	10	1.476	0.007	0.036	Co-occurrence
56	HDAC7	FCHO1	1709	78	105	12	1.324	0.007	0.037	Co-occurrence

Supplementary Table 2.

The list of 56 genes that are upregulated by HDAC1/7-SE in vitro and correlate with in vivo human breast cancer HDAC7 over-expression in the cBioPortal METABRIC dataset. The p-values were computed using Log Rank test, and the q-values were computed using the Benjamini-Hochberg false discovery rate procedure.

A. TOP 10 CURATED GENE SETS (C2, n=6,290)	Description	# Genes in Overlap (k)	p-value	FDR q-value
**SENESE_HDAC1_TARGETS_DN [266]**	Genes down-regulated in U2OS cells (osteosarcoma) upon knockdown of HDAC1 [GeneID=3065] by RNAi.	13	2.66 e^{-12}	1.01 e^{-8}
DODD_NASOPHARYNGEAL_CARCINOMA_UP [1823]	Genes up-regulated in nasopharyngeal carcinoma (NPC) compared to the normal tissue.	27	1.03 e^{-11}	1.96 e^{-8}
KEGG_PATHWAYS_IN_CANCER [325]	Pathways in cancer	13	3.26 e^{-11}	4.14 e^{-8}
**SENESE_HDAC1_AND_HDAC2_TARGETS_DN [237]**	Genes down-regulated in U2OS cells upon knockdown of both HDAC1 and HDAC2 [GeneID=3065;3066] by RNAi.	11	2.37 e^{-10}	2.25 e^{-7}
BLALOCK_ALZHEIMERS_DISEASE_UP [1673]	Genes up-regulated in brain from patients with Alzheimer's disease.	24	3.3 e^{-10}	2.51 e^{-7}
KEGG_FOCAL_ADHESION [199]	Focal adhesion	10	7.42 e^{-10}	4.72 e^{-7}
WONG_ADULT_TISSUE_STEM_MODULE [719]	The 'adult tissue stem' module: genes coordinately up-regulated in a compendium of adult tissue stem cells.	16	9.15 e^{-10}	4.98 e^{-7}
KOINUMA_TARGETS_OF_SMAD2_OR_SMAD3 [846]	Genes with promoters occupied by SMAD2/3 [GeneID=4087, 4088] in HaCaT cells (keratinocyte) according to a ChIP-chip analysis.	17	1.22 e^{-9}	5.83 e^{-7}
WP_DNA_DAMAGE_RESPONSE_ONLY_ATM_DEPENDENT [114]	DNA Damage Response (only ATM dependent)	8	2.85 e^{-9}	1.21 e^{-6}
JINESH_BLEBBISHIELD_TO_IMMUNE_CELL_FUSION_PBSHMS_UP [396]	Genes Upregulated in PBSHMS (RT4 blebbishield-to-immune cell fusion)	12	4.45 e^{-9}	1.68 e

B. TOP 10 HALLMARK GENE SETS (H, n=50)	Description	# Genes in Overlap (k)	p-value	FDR q-value
HALLMARK_EPITHELIAL_MESENCHYMAL_TRANSITION [200]	Genes defining epithelial-mesenchymal transition, as in wound healing, fibrosis and metastasis.	8	2.28 e^{-7}	1.14 e^{-5}
HALLMARK_KRAS_SIGNALING_DN [200]	Genes down-regulated by KRAS activation.	7	3.24 e^{-6}	8.1 e^{-5}
HALLMARK_UV_RESPONSE_DN [144]	Genes down-regulated in response to ultraviolet (UV) radiation.	6	6.19 e^{-6}	1.03 e^{-4}
HALLMARK_APOPTOSIS [161]	Genes mediating programmed cell death (apoptosis) by activation of caspases.	5	1.52 e^{-4}	1.9 e^{-3}
HALLMARK_WNT_BETA_CATENIN_SIGNALING [42]	Genes up-regulated by activation of WNT signaling through accumulation of beta catenin CTNNB1 [GeneID=1499].	3	3.06 e^{-4}	2.07 e^{-3}
HALLMARK_ALLOGRAFT_REJECTION [200]	Genes up-regulated during transplant rejection.	5	4.14 e^{-4}	2.07 e^{-3}
HALLMARK_HYPOXIA [200]	Genes up-regulated in response to low oxygen levels (hypoxia).	5	4.14 e^{-4}	2.07 e^{-3}
HALLMARK_MYOGENESIS [200]	Genes involved in development of skeletal muscle (myogenesis).	5	4.14 e^{-4}	2.07 e^{-3}
HALLMARK_P53_PATHWAY [200]	Genes involved in p53 pathways and networks.	5	4.14 e^{-4}	2.07 e^{-3}
HALLMARK_XENOBIOTIC_METABOLISM [200]	Genes encoding proteins involved in processing of drugs and other xenobiotics.	5	4.14 e^{-4}	2.07 e^{-3}

C. TOP 10 CURATED GENE SETS (C3-8, n= 16633)	Description	# Genes in Overlap (k)	p-value	FDR q-value
**ZNF92_TARGET_GENES [1440]**	Genes containing one or more binding sites for UniProt:Q03936 (ZNF92) in their promoter regions (TSS -1000,+100 bp) as identified by GTRD version 20.06 ChIP-seq harmonization.	29	6.5 e-16	1.08 e-11
GOBP_NEUROGENESIS [1613]	Generation of cells within the nervous system. [GO_REF:0000021	28	8.74 e-14	7.27 e-10
GOBP_POSITIVE_REGULATION_OF_CELL_POPULATION_PROLIFERATION [941]	Any process that activates or increases the rate or extent of cell proliferation. [GOC:go_curators]	22	1.73 e-13	9.57 e-10
GOBP_BIOLOGICAL_ADHESION [1481]	The attachment of a cell or organism to a substrate, another cell, or other organism, includes intracellular attachment	26	6.03 e-13	2.51 e-9
GOBP_LOCOMOTION [1975]	Self-propelled movement of a cell or organism from one location to another. [GOC:dgh]	29	1.86 e-12	6.19 e-9
GOBP_CELL_CELL_SIGNALING [1672]	Any process that mediates the transfer of information from one cell to another, including via soluble ligands, via cell adhesion molecules and via gap junctions. [GOC:dos, GOC:mah]	26	9.08 e-12	2.52 e-8
GOBP_REGULATION_OF_CELL_ADHESION [732]	Any process that modulates the frequency, rate or extent of attachment of a cell to another cell or to the extracellular matrix.	18	1.47 e-11	3.5 e-8
GOBP_TAXIS [644]	The directed movement of a motile cell or organism in response to an external stimulus. [GOC:jl, ISBN:0192801023]	17	1.89 e-11	3.92 e-8
GOBP_CELL_MIGRATION [1602]	The controlled self-propelled movement of a cell from one site to a destination guided by molecular cues. [GOC:cjm, GOC:dph, GOC:ems, GOC:pf, Wikipedia:Cell_migration]	25	2.26 e-11	4.17 e-8
ZNF768_TARGET_GENES [1346]	Genes containing one or more binding sites for UniProt:Q9H5H4 (ZNF768) in their promoter regions (TSS -1000,+100 bp) as identified by GTRD v 20.06 ChIP-seq	23	2.69 e-11	4.47 e-8

Supplementary Table 3.

Gene Set Enrichment Analysis of 125 HDAC1/7-SE upregulated genes. The p values computed using Hypergeometric test.

	Cancer type	TCGA PanCancer Dataset	No. of samples in TCGA
1	Breast cancer	Breast Invasive Carcinoma (BRCA)	1075
2	Cervical cancer	Cervical Carcinoma (CESC)	291
3	Colorectal cancer	Colon Adenocarcinoma (COAD)	438
4		Rectum Adenocarcinoma (READ)	159
5	Endometrial cancer	Uterine Corpus Endometrial Carcinoma (UCEC)	541
6	Glioma	Glioblastoma Multiforme (GBM)	153
7	Head and neck cancer	Head and Neck Squamous Cell Carcinoma (HNSC)	499
8	Liver cancer	Liver Hepatocellular Carcinoma (LIHC)	365
9	Lung cancer	Lung Adenocarcinoma (LUAD)	500
10		Lung Squamous Cell Carcinoma (LUSC)	494
11	Melanoma	Skin Cuteneous Melanoma (SKCM)	102
12	Ovarian cancer	Ovary Serous Cystadenocarcinoma (OV)	373
13	Pancreatic cancer	Pancreatic Adenocarcinoma (PAAD)	176
14	Prostate cancer	Prostate Adenocarcinoma (PRAD)	494
15	Renal cancer	Kidney Chromophobe (KICH)	64
16		Kidney Renal Clear Cell Carcinoma (KIRC)	528
17		Kidney Renal Papillary Cell Carcinoma (KIRP)	285
18	Stomach cancer	Stomach Adenocarcinoma (STAD)	354
19	Testis cancer	Testicular Germ Cell Tumor (TGCT)	134
20	Thyroid cancer	Thyroid Carcinoma (THCA)	501
21	Urothelial cancer	Bladder Urothelial Carcinoma (BLCA)	406
	TOTAL		7932

Supplementary Table 4.

List of tumor types in the Human Protein Atlas (HPA) PanCancer dataset. The RNA-seq data includes 17 cancer types representing 21 cancer subtypes. The TCGA RNA-seq data was mapped using the Ensembl gene id available from TCGA, and the FPKMs (number Fragments Per Kilobase of exon per Million reads) for each gene were subsequently used for quantification of expression with a detection threshold of 1 FPKM.

	Abbreviation	Name		Abbreviation	Name
1	ACC	Adrenocortical carcinoma	19	LUAD	Lung adenocarcinoma
2	BLCA	Bladder Urothelial Carcinoma	20	LUSC	Lung squamous cell carcinoma
3	BRCA	Breast invasive carcinoma	21	MESO	Mesothelioma
4	CESC	Cervical squamous cell & adenocarcinoma	22	MISC	Miscellaneous
5	CHOL	Cholangiocarcinoma	23	OV	Ovarian serous carcinoma
6	CNTL	Controls	24	PAAD	Pancreatic adenocarcinoma
7	COAD	Colon adenocarcinoma	25	PCPG	Pheochromocytoma/Paraganglioma
8	DLBC	Diffuse Large B-cell Lymphoma	26	PRAD	Prostate adenocarcinoma
9	ESCA	Esophageal carcinoma	27	READ	Rectum adenocarcinoma
10	FPPP	FFPE Pilot Phase II	28	SARC	Sarcoma
11	GBM	Glioblastoma multiforme	29	SKCM	Skin Cutaneous Melanoma
12	HNSC	Head and Neck squamous cell carcinoma	30	STAD	Stomach adenocarcinoma
13	KICH	Kidney Chromophobe	31	TGCT	Testicular Germ Cell Tumors
14	KIRC	Kidney renal clear cell carcinoma	32	THCA	Thyroid carcinoma
15	KIRP	Kidney renal papillary cell carcinoma	33	THYM	Thymoma
16	LAML	Acute Myeloid Leukemia	34	UCEC	Uterine Corpus Endometrial Carcinoma
17	LCML	Chronic Myelogenous Leukemia	35	UCS	Uterine Carcinosarcoma
18	LGG	Brain Lower Grade Glioma	36	UVM	Uveal Melanoma
19	LIHC	Liver hepatocellular carcinoma			

Supplementary Table 5.

The list of 36 tumor types in the TCGA PanCancer dataset with RNA-seq data in cBioPortal

#	Entrez ID	Gene Symbol	Gene Description
1	9289	***ADGRG1 (GRP56)***	adhesion G protein-coupled receptor G1 [Source:HGNC Symbol;Acc:HGNC:4512]
2	27092	***CACNG4***	calcium voltage-gated channel auxiliary subunit gamma 4 [Source:HGNC Symbol;Acc:HGNC:1408]
3	26112	***CCDC69***	coiled-coil domain containing 69 [Source:HGNC Symbol;Acc:HGNC:24487]
4	6376	***CX3CL1***	C-X3-C motif chemokine ligand 1 [Source:HGNC Symbol;Acc:HGNC:10647]
5	84929	***FIBCD1***	fibrinogen C domain containing 1 [Source:HGNC Symbol;Acc:HGNC:25922]
6	81544	***GDPD5***	glycerophosphodiester phosphodiesterase domain containing 5 [Source:HGNC Symbol;Acc:HGNC:28804]
7	3488	***IGFBP5***	insulin like growth factor binding protein 5 [Source:HGNC Symbol;Acc:HGNC:5474]
8	4135	***MAP6***	microtubule associated protein 6 [Source:HGNC Symbol;Acc:HGNC:6868]
9	56241	***SUSD2***	sushi domain containing 2 [Source:HGNC Symbol;Acc:HGNC:30667]
10	80325	***ABTB1***	ankyrin repeat and BTB domain containing 1 [Source:HGNC Symbol;Acc:HGNC:18275]
11	55653	***BCAS4***	breast carcinoma amplified sequence 4 [Source:HGNC Symbol;Acc:HGNC:14367]
12	149428	***BNIPL***	BCL2 interacting protein like [Source:HGNC Symbol;Acc:HGNC:16976]
13	91653	***BOC***	"BOC cell adhesion associated, oncogene regulated [Source:HGNC Symbol;Acc:HGNC:17173]"
14	894	***CCND2***	cyclin D2 [Source:HGNC Symbol;Acc:HGNC:1583]
15	51200	***CPA4***	carboxypeptidase A4 [Source:HGNC Symbol;Acc:HGNC:15740]
16	9696	***CROCC***	"ciliary rootlet coiled-coil, rootletin [Source:HGNC Symbol;Acc:HGNC:21299]"
17	1445	***CSK***	C-terminal Src kinase [Source:HGNC Symbol;Acc:HGNC:2444]
18	23399	***CTDNEP1***	CTD nuclear envelope phosphatase 1 [Source:HGNC Symbol;Acc:HGNC:19085]
19	1523	***CUX1***	cut like homeobox 1 [Source:HGNC Symbol;Acc:HGNC:2557]
20	29785	***CYP2S1***	cytochrome P450 family 2 subfamily S member 1 [Source:HGNC Symbol;Acc:HGNC:15654]
21	50619	***DEF6***	DEF6 guanine nucleotide exchange factor [Source:HGNC Symbol;Acc:HGNC:2760]
22	414918	***DENND6B***	DENN domain containing 6B [Source:HGNC Symbol;Acc:HGNC:32690]
23	27122	***DKK3***	dickkopf WNT signaling pathway inhibitor 3 [Source:HGNC Symbol;Acc:HGNC:2893]
24	1891	***ECH1***	enoyl-CoA hydratase 1 [Source:HGNC Symbol;Acc:HGNC:3149]
25	2027	***ENO3***	enolase 3 [Source:HGNC Symbol;Acc:HGNC:3354]
26	2049	***EPHB3***	EPH receptor B3 [Source:HGNC Symbol;Acc:HGNC:3394]
27	23149	***FCHO1***	FCH and mu domain containing endocytic adaptor 1 [Acc:HGNC:29002]
28	2246	***FGF1***	fibroblast growth factor 1 [Source:HGNC Symbol;Acc:HGNC:3665]
29	2535	***FZD2***	frizzled class receptor 2 [Source:HGNC Symbol;Acc:HGNC:4040]
30	79885	***HDAC11***	histone deacetylase 11 [Source:HGNC Symbol;Acc:HGNC:19086]
31	3399	***ID3***	"inhibitor of DNA binding 3, HLH protein [Source:HGNC Symbol;Acc:HGNC:5362]"
32	389792	***IER5L***	immediate early response 5 like [Source:HGNC Symbol;Acc:HGNC:23679]
33	3569	***IL6***	interleukin 6 [Source:HGNC Symbol;Acc:HGNC:6018]
34	3855	***KRT7***	keratin 7 [Source:HGNC Symbol;Acc:HGNC:6445]
35	3911	***LAMA5***	laminin subunit alpha 5 [Source:HGNC Symbol;Acc:HGNC:6485]
36	3985	***LIMK2***	LIM domain kinase 2 [Source:HGNC Symbol;Acc:HGNC:6614]
37	4016	***LOXL1***	lysyl oxidase like 1 [Source:HGNC Symbol;Acc:HGNC:6665]
38	4017	***LOXL2***	lysyl oxidase like 2 [Source:HGNC Symbol;Acc:HGNC:6666]
39	4035	***LRP1***	LDL receptor related protein 1 [Source:HGNC Symbol;Acc:HGNC:6692]
40	4151	***MB***	myoglobin [Source:HGNC Symbol;Acc:HGNC:6915]
41	4245	***MGAT1***	"alpha-1,3-mannosyl-glycoprotein 2-beta-N-acetylglucosaminyltransferase [Acc:HGNC:7044]"
42	4430	***MYO1B***	myosin IB [Source:HGNC Symbol;Acc:HGNC:7596]
43	4665	***NAB2***	NGFI-A binding protein 2 [Source:HGNC Symbol;Acc:HGNC:7627]
44	5097	***PCDH1***	protocadherin 1 [Source:HGNC Symbol;Acc:HGNC:8655]
45	9124	***PDLIM1***	PDZ and LIM domain 1 [Source:HGNC Symbol;Acc:HGNC:2067]
46	8398	***PLA2G6***	phospholipase A2 group VI [Source:HGNC Symbol;Acc:HGNC:9039]
47	57580	***PREX1***	"phosphatidylinositol-3,4,5-trisphosphate dependent Rac exchange factor 1 [Acc:HGNC:32594]"
48	25766	***PRPF40B***	pre-mRNA processing factor 40 homolog B [Source:HGNC Symbol;Acc:HGNC:25031]
49	5875	***RABGGTA***	Rab geranylgeranyltransferase subunit alpha [Source:HGNC Symbol;Acc:HGNC:9795]
50	6277	***S100A6***	S100 calcium binding protein A6 [Source:HGNC Symbol;Acc:HGNC:10496]
51	6337	***SCNN1A***	sodium channel epithelial 1 subunit alpha [Source:HGNC Symbol;Acc:HGNC:10599]
52	6464	***SHC1***	SHC adaptor protein 1 [Source:HGNC Symbol;Acc:HGNC:10840]
53	92799	***SHKBP1***	SH3KBP1 binding protein 1 [Source:HGNC Symbol;Acc:HGNC:19214]
54	9751	***SNPH***	syntaphilin [Source:HGNC Symbol;Acc:HGNC:15931]
55	115572	***TENT5B***	terminal nucleotidyltransferase 5B [Source:HGNC Symbol;Acc:HGNC:28273]
56	7057	***THBS1***	thrombospondin 1 [Source:HGNC Symbol;Acc:HGNC:11785]
57	79639	***TMEM53***	transmembrane protein 53 [Source:HGNC Symbol;Acc:HGNC:26186]
58	7433	***VIPR1***	vasoactive intestinal peptide receptor 1 [Source:HGNC Symbol;Acc:HGNC:12694]
59	80326	***WNT10A***	Wnt family member 10A [Source:HGNC Symbol;Acc:HGNC:13829]
60	7508	***XPC***	"XPC complex subunit, DNA damage recognition and repair factor [Acc:HGNC:12816]"

Supplementary Table 6.

The list of ET-60 signature genes

ET-9 non-significant clinical associations	Statistical Test	p-Value	q-Value
AJCC Disease Stage	Chi-squared Test	0.349	0.509
AJCC Lymph Node Stage	Chi-squared Test	0.797	0.853
AJCC Metastasis Stage	Chi-squared Test	0.0623	0.145
AJCC Tumor Stage	Chi-squared Test	0.413	0.589
Aneuploidy Score	Wilcoxon Test	0.158	0.297
Diagnosis Age	Wilcoxon Test	0.515	0.64
Ethnicity Category	Chi-squared Test	0.335	0.496
Fraction Genome Altered	Wilcoxon Test	0.0347	0.111
Mutation Count	Wilcoxon Test	0.0121	0.0701
Primary Lymph Node Presentation Assessment	Chi-squared Test	0.424	0.589
Prior Diagnosis	Chi-squared Test	0.0562	0.142
Race Category	Chi-squared Test	0.0205	0.0839
Radiation Therapy	Chi-squared Test	0.874	0.885
Winter Hypoxia Score	Wilcoxon Test	0.013	0.0701

Supplementary Table 7.
Multivariate analysis of ET-9 in the TCGA Invasive Breast Cancer datasets. Analysis carried out using cBioPortal online tools, in the TCGA Breast Invasive Carcinoma dataset, complete samples (n=1,084).

A.

Pathways	Signature Tested
ET-9 Signature (9 genes)	ADGRG1 (GRP56), FIBCD1, GDPD5, ABCC2, SUSD2, CACNG4, CX3CL1, IGFBP5, MAP6, CCDC69
Cell Cycle (34 genes)	RB1 RBL1 RBL2 CCNA1 CCNB1 CDK1 CCNE1 CDK2 CDC25A CCND1 CDK4 CDK6 CCND2 CDKN2A CDKN2B MYC CDKN1A CDKN1B E2F1 E2F2 E2F3 E2F4 E2F5 E2F6 E2F7 E2F8 SRC JAK1 JAK2 STAT1 STAT2 STAT3 STAT5A STAT5B
P53 (6 genes)	TP53 MDM2 MDM4 CDKN2A CDKN2B TP53BP1
PI3K-AKT-mTOR signaling (17 genes)	PIK3CA PIK3R1 PIK3R2 PTEN PDPK1 AKT1 AKT2 FOXO1 FOXO3 MTOR RICTOR TSC1 TSC2 RHEB AKT1S1 RPTOR MLST8
Notch Signaling (55 genes)	ADAM10 ADAM17 APH1A APH1B ARRDC1 CIR1 CTBP1 CTBP2 CUL1 DLL1 DLL3 DLL4 DTX1 DTX2 DTX3 DTX3L DTX4 EP300 FBXW7 HDAC1 HDAC2 HES1 HES5 HEYL ITCH JAG1 JAG2 KDM5A LFNG MAML1 MAML2 MAML3 MFNG NCOR2 NCSTN NOTCH1 NOTCH2 NOTCH3 NOTCH4 NRARP NUMB NUMBL PSEN1 PSEN2 PSENEN RBPJ RBPJL RFNG SNW1 SPEN HES2 HES4 HES7 HEY1 HEY2
Ras-Raf-MEK-Erk/JNK signaling (26 genes)	KRAS HRAS BRAF RAF1 MAP3K1 MAP3K2 MAP3K3 MAP3K4 MAP3K5 MAP2K1 MAP2K2 MAP2K3 MAP2K4 MAP2K5 MAPK1 MAPK3 MAPK4 MAPK6 MAPK7 MAPK8 MAPK9 MAPK12 MAPK14 DAB2 RASSF1 RAB25
TGF-B Pathway (43 genes)	TGFB1 TGFB2 TGFB3 TGFBR1 TGFBR2 TGFBR3 BMP2 BMP3 BMP4 BMP5 BMP6 BMP7 GDF2 BMP10 BMP15 BMPR1A BMPR1B BMPR2 ACVR1 ACVR1B ACVR1C ACVR2A ACVR2B ACVRL1 Nodal GDF1 GDF11 INHA INHBA INHBB INHBC INHBE SMAD2 SMAD3 SMAD1 SMAD5 SMAD4 SMAD9 SMAD6 SMAD7 SPTBN1 TGFBRAP1 ZFYVE9
Oncotype Dx (21 genes)	CTSV, GRB7, ERBB2, ESR1, PGR, BCL2, SCUBE2, GSTM1, BAG1, CD68, ACTB, GAPDH, GUS, RPLPO, TFRC

B.

Comparison of Prognostic Signatures	TCGA (n=1,084)				METABRIC (n=1,904)	
	Overall	Progression free	Disease specific	Disease Free	Overall	Relapse Free
ET-9 Signature (9 genes)	**p=1.64E-04**	**p=2.31E-03**	**p=1.56E-05**	**p=1.02E-02**	**p=5.07E-03**	**p=6.12E-03**
Prosignia (PAM50, 50 genes)	p=0.52	p=0.89	p=0.59	p=0.84	**p=0.03**	**p=5.82E-03**
Oncotype Dx (21 genes)	p=0.66	p=0.97	p=0.88	p=0.86	p=0.09	**p=3.25E-03**
Mammaprint (70 genes)	p=0.09	p=0.20	**p=3.03E-02**	p=0.19	**p=9.68E-03**	p=0.47
Cell Cycle Control (34 genes)	p=0.26	p=0.26	p=0.55	**p=3.8E-02**	p=0.30	p=0.21
p53 (6 genes)	p=0.77	P=0.85	p=0.66	P=0.90	p=0.74	**p=1.08E-02**
PI3K-AKT-mTOR signaling (17 genes)	p=0.29	p=0.29	p=0.47	p=0.61	p=0.059	p=0.45
Notch Signaling (55 genes)	p=0.79	p=0.46	p=0.18	p=0.31	p=0.95	p=0.45
Ras-Raf-MEK-Erk/JNK signaling (26 genes)	p=0.10	p=0.60	p=0.25	p=0.63	p=0.07	p=0.26
TGF-B Pathway (43 genes)	p=0.36	p=0.22	p=0.63	p=0.19	p=0.35	p=0.27

Supplementary Table 8.
Survival analysis carried out using cBioPortal online tools, in the TCGA Breast Invasive Carcinoma dataset, complete samples (n=1,084) and Metabric dataset (n=1,094). The p-values were computed using Log Rank test.

A.

	**Collections**	**Gene stets that overlap with 70-gene signature (Mammaprint)**	**# Gene Sets in Collections**	**# Genes in Universe (N)**	
	GO	20	14998	40312	
	Gene Set Name [# Genes (K)]	Description	# Genes in Overlap (k)	p-value	FDR q-value
1	**GOBP_CELL_DIVISION [600]**	The process resulting in division and partitioning of components of a cell to form more cells [GOC:di, GOC:go_curators, GOC:pr]	10	$2.26 e^{-8}$	$2.29 e^{-4}$
2	GOBP_POSITIVE_REGULATION_OF_SIGNALING [1725]	Any process that activates, maintains or increases the frequency, rate or extent of a signaling process. [GOC:mtg_signal]	15	$3.06 e^{-8}$	$2.29 e^{-4}$
3	**GOBP_MITOTIC_CELL_CYCLE [1032]**	Progression through the phases of the mitotic cell cycle. [GOC:mah, ISBN:0815316194, Reactome:69278]	11	$3.81 e^{-7}$	$1.91 e^{-3}$
4	**GOBP_CELL_CYCLE_PROCESS [1415]**	The cellular process that ensures successive accurate and complete genome replication and chromosome segregation. [GOC:isa_complete, GOC:mtg_cell_cycle]	12	$1.18 e^{-6}$	$4.44 e^{-3}$
5	**GOMF_GROWTH_FACTOR_BINDING [137]**	Interacting selectively and non-covalently with any growth factor, proteins or polypeptides that stimulate a cell or organism to grow or proliferate. [GOC:curators]	5	$2.15 e^{-6}$	$6.45 e^{-3}$
6	**GOBP_CELL_CYCLE [1872]**	The progression of biochemical and morphological phases and events that occur in a cell during successive cell replication or nuclear replication events. [GOC:go_curators, GOC:mtg_cell_cycle]	13	$3.64 e^{-6}$	$7.15 e^{-3}$
7	**GOBP_DNA_DEPENDENT_DNA_REPLICATION [157]**	A DNA replication process that uses parental DNA as a template for the DNA-dependent DNA polymerases that synthesize the new strands. [GOC:mah, ISBN:0198506732]	5	$4.19 e^{-6}$	$7.15 e^{-3}$
8	GOMF_RIBONUCLEOTIDE_BINDING [1898]	Interacting selectively and non-covalently with a ribonucleotide, any compound consisting of a ribonucleoside that is esterified with (ortho)phosphate or an oligophosphate at any hydroxyl group on the ribose moiety. [GOC:mah]	13	$4.24 e^{-6}$	$7.15 e^{-3}$
9	**GOBP_DNA_REPLICATION [280]**	The cellular metabolic process in which a cell duplicates one or more molecules of DNA. [GOC:mah]	6	$4.29 e^{-6}$	$7.15 e^{-3}$
10	GOBP_CIRCULATORY_SYSTEM_DEVELOPMENT [1140]	The process whose specific outcome is the progression of the circulatory system over time, from its formation to the mature structure. [GOC:mah, UBERON:0001009]	10	$7.66 e^{-6}$	$1.15 e^{-2}$

B.

	Collections	Gene stets that overlap with PAM-50 Signature	# Gene Sets in Collections	# Genes in Universe (N)	
	GO	20	10185	40312	
	Gene Set Name [# Genes (K)]	Description	# Genes in Overlap (k)	p-value	FDR q-value
1	**GOBP_MITOTIC_CELL_CYCLE [1032]**	Progression through the phases of the mitotic cell cycle.[GOC:mah, ISBN:0815316194, Reactome:69278]	24	$3.12 e^{-25}$	$3.18 e^{-21}$
2	**GOBP_CELL_CYCLE [1872]**	The progression of biochemical and morphological phases and events that occur in a cell during successive cell replication or nuclear replication events [GOC:go_curators, GOC:mtg_cell_cycle]	28	$1.27 e^{-24}$	$6.48 e^{-21}$
3	**GOBP_CELL_CYCLE_PROCESS [1415]**	The cellular process that ensures successive accurate and complete genome replication and chromosome segregation. [GOC:isa_complete, GOC:mtg_cell_cycle]	24	$5.13 e^{-22}$	$1.74 e^{-18}$
4	**GOBP_REGULATION_OF_CELL_CYCLE [1211]**	Any process that modulates the rate or extent of progression through the cell cycle. [GOC:ai, GOC:dph, GOC:tb]	20	$6.15 e^{-18}$	$1.57 e^{-14}$
5	**GOBP_CELL_CYCLE_PHASE_TRANSITION [641]**	The cell cycle process by which a cell commits to entering the next cell cycle phase. [GOC:mtg_cell_cycle]	16	$4.14 e^{-17}$	$8.44 e^{-14}$
6	**GOBP_CELL_DIVISION [600]**	The process resulting in division and partitioning of components of a cell to form more cells [GOC:di, GOC:go_curators, GOC:pr]	15	$4.56 e^{-16}$	$7.75 e^{-13}$
7	GOBP_ORGANELLE_FISSION [486]	The creation of two or more organelles by division of one organelle. [GOC:jid]	14	$7.18 e^{-16}$	$1.04 e^{-12}$
8	**GOBP_MITOTIC_NUCLEAR_DIVISION [296]**	A mitotic cell cycle process comprising the steps by which the nucleus of a eukaryotic cell divides [ISBN:0198547684]	12	$1.86 e^{-15}$	$2.16 e^{-12}$
9	**GOBP_REGULATION_OF_CHROMOSOME_SEGREGATION [89]**	Any process that modulates the frequency, rate or extent of chromosome segregation, [GOC:ai]	9	$1.91 e^{-15}$	$2.16 e^{-12}$
10	**GOBP_CHROMOSOME_SEGREGATION [337]**	Chromosome segregation [GOC:jl, GOC:mah, GOC:mtg_cell_cycle, GOC:vw]	12	$8.75 e^{-15}$	$8.91 e^{-12}$

Supplementary Table 9.

The top ten gene sets that overlap with the Mammaprint and PAM-50 signatures in GSEA. The p values in GSEA are computed using Hypergeometric test.

References

1 Curtis, C. *et al.* The genomic and transcriptomic architecture of 2,000 breast tumours reveals novel subgroups. *Nature* **486**, 346-352, doi:10.1038/nature10983 (2012).

2 Pereira, B. *et al.* The somatic mutation profiles of 2,433 breast cancers refines their genomic and transcriptomic landscapes. *Nat Commun* **7**, 11479, doi:10.1038/ncomms11479 (2016).

3 Gao, J. *et al.* Integrative analysis of complex cancer genomics and clinical profiles using the cBioPortal. *Sci Signal* **6**, pl1, doi:10.1126/scisignal.2004088 (2013).

4 Aguirre-Gamboa, R. *et al.* SurvExpress: an online biomarker validation tool and database for cancer gene expression data using survival analysis. *PLoS One* **8**, e74250, doi:10.1371/journal.pone.0074250 (2013).

5 Lanczky, A. & Gyorffy, B. Web-Based Survival Analysis Tool Tailored for Medical Research (KMplot): Development and Implementation. *J Med Internet Res* **23**, e27633, doi:10.2196/27633 (2021).

6 Ghantous, Y. *et al.* A robust and interpretable gene signature for predicting the lymph node status of primary T1/T2 oral cavity squamous cell carcinoma. *Int J Cancer* **150**, 450-460, doi:10.1002/ijc.33828 (2022).

7 Marchionni, L., Afsari, B., Geman, D. & Leek, J. T. A simple and reproducible breast cancer prognostic test. *BMC Genomics* **14**, 336, doi:10.1186/1471-2164-14-336 (2013).
